# Dose-dependent association of hyperoxia and decreased favorable outcomes in mechanically ventilated patients with traumatic brain injury, a retrospective cohort study

**DOI:** 10.1007/s00068-024-02730-5

**Published:** 2025-01-24

**Authors:** Louisa Telsche Lalla, Patrick Czorlich, Marlene Fischer, Nils Schweingruber, Christopher Cramer, Karl-Heinz Frosch, Jens Gempt, Stefan Kluge, Jörn Grensemann

**Affiliations:** 1 Department of Intensive Care Medicine, University Medical Center Hamburg-Eppendorf, Martinistraße 52, 20246 Hamburg, Germany; 2 Department of Neurosurgery, University Medical Center Hamburg-Eppendorf, Martinistraße 52, 20246 Hamburg, Germany; 3 Department of Neurology, University Medical Center Hamburg-Eppendorf, Martinistraße 52, 20246 Hamburg, Germany; 4 Department of Trauma Surgery and Orthopedics, University Medical Center Hamburg-Eppendorf, Martinistraße 52, 20246 Hamburg, Germany

**Keywords:** Traumatic brain injury, Neurocritical care, Hyperoxia, PaO_2_, Oxygen exposure, Oxygen toxicity

## Abstract

**Purpose:**

In patients with traumatic brain injury (TBI), adequate oxygenation is crucial to optimize survival and neurological outcome. However, supranormal oxygen partial pressure (PaO_2_) only leads to minor increase in cerebral oxygen delivery but can cause numerous pathophysiological disturbances. Therefore, we aimed to study effects of hyperoxia on patient outcome and identify optimum PaO_2_ ranges.

**Methods:**

This retrospective, single-center cohort study included TBI patients receiving mechanical ventilation for ≥ 72 h. Time-weighted mean PaO_2_ and integrals above thresholds of 80, 100, 120, and 150 mmHg were calculated over periods of 1, 3, 7, and 14 days. The effects on in-hospital mortality and favorable functional outcome defined as Glasgow Outcome Scale (GOS) ≥ 4 were explored at discharge and after 3–6 months.

**Results:**

From 01/2013 until 12/2021, 290 patients fulfilled the inclusion criteria. Hyperoxia was dose-dependently associated with a worsened functional outcome 3–6 months post-injury. Regarding the first 24 h, odds ratios were 0.959 (95% confidence intervals: 0.932–0.990; *p* = 0.009) for time-weighted mean PaO_2_ and 0.955 (0.923–0.988; *p* = 0.008), 0.939 (0.897–0.982; *p* = 0.006), 0.923 (0.871–0.978; *p* = 0.007) and 0.922 (0.858–0.992; *p* = 0.029) per mmHg above 80, 100, 120 and 150 mmHg, respectively. For exposure within 72 h, odds ratios were 0.897 (0.819–0.983; *p* = 0.020), 0.842 (0.738–0.961; *p* = 0.011) and 0.832 (0.705–0.981; *p* = 0.029) per mmHg per day over 100, 120 and 150 mmHg, respectively. No significant association could be established between PaO_2_-exposure and in-hospital mortality, GOS at discharge or the 7- and 14-day periods.

**Conclusion:**

In this cohort, hyperoxia within 72 h after admission was dose-dependently associated with an unfavorable neurological outcome after 3–6 months.

**Supplementary Information:**

The online version contains supplementary material available at 10.1007/s00068-024-02730-5.

## Introduction

Providing adequate oxygen delivery is lifesaving in critically ill multiple trauma patients who often suffer from a concomitant traumatic brain injury (TBI). Hypoxia is a proven risk factor for mortality and morbidity in TBI patients and its prevention is essential to avoid further brain damage [1]. However, this may lead to excessive oxygen supplementation and inadvertent hyperoxia. Oxygen delivery depends on various factors such as cardiac output, hemoglobin levels, and particularly on arterial oxygen partial pressure (PaO_2_). In the blood, oxygen is mainly transported bound to hemoglobin with a sigmoidal binding curve and only a negligible amount is physically dissolved. Above a PaO_2_ of 80 mmHg, corresponding to an oxygen saturation (SpO_2_) of approximately 96%, only little additional oxygen may be bound to hemoglobin [2]. However, a further increase of PaO_2_ may increase the formation of reactive oxygen species (ROS) promoting cell damage by apoptosis or even necrosis [3]. Despite that, the current German guideline for management of TBI in adult patients recommends an oxygen saturation above 90% without specifying an upper threshold [4]. Several clinical studies have shown detrimental effects for hyperoxia, even impaired neurological outcome [5] and increased mortality [6]. Nevertheless, there is a lack of appropriate trials examining the consequences of prolonged hyperoxia in patients with TBI, and, to date, the optimal oxygen target range for TBI patients remains unknown. Previous retrospective studies evaluated mainly the initial measurement or the first day after admission [7–15], and a randomized prospective trial was terminated due to slow recruitment [16]. Recently, our working group could show an association between a PaO_2_-range of 78 to 85 mmHg and the lowest mortality and the most favorable outcomes after three months in patients with aneurysmal subarachnoid hemorrhage and could demonstrate a dose-dependent toxicity [17]. We have postulated a certain threshold for oxygen partial pressure (PaO_2_) beyond which additional oxygen supplementation becomes an independent risk factor for patient well-being. To define potential optimal oxygen target ranges in mechanically ventilated TBI patients, we aimed to determine the association between oxygen exposure and clinical outcome parameters in this exploratory, retrospective cohort study.

## Methods

### Ethics

The retrospective and anonymized data collection and analysis were conducted in accordance with local government law (HmbKHG. § 12) without the requirement for approval or informed consent. The study was performed in accordance with the ethical standards as laid down in the 1964 Declaration of Helsinki and its later amendments or comparable ethical standards.

### Study Design

This retrospective single-center cohort study was aiming to explore the association between long-term oxygen exposure and clinical outcome in mechanically ventilated patients with traumatic brain injury. The study is compliant with the Strengthening the Reporting of Observational Studies in Epidemiology (STROBE) reporting guidelines.

### Setting and population

The study was jointly conducted at the Department of Intensive Care, Department of Neurosurgery, Department of Neurology, and Department of Trauma Surgery and Orthopedics at the University Medical Center, Hamburg-Eppendorf, Germany. All patients discharged from the intensive care unit (ICU) between January 2013 and December 2021 were reviewed. Patients above the age of 15 years were included if they were admitted to the ICU for recent TBI and mechanically ventilated for at least 72 h. Patients with multiple injuries were excluded from the study if the Abbreviated Injury Scale (AIS) for the head was lower than the AIS score for any other body region, as well as patients with an isolated epidural hematoma.

### Management of traumatic brain injury

According to hospital protocols, patients with TBI received an intracranial pressure (ICP) probe. An ICP lower than 20 mmHg was targeted, which was achieved by (a) adjusting the minute ventilation, targeting an arterial partial pressure of carbon dioxide of 35 mmHg, (b) hyperosmolar therapy, i.e. sodium chloride or mannitol, (c) head-up positioning of 15° to 30°, and (d) sedation. Sedation was commenced with propofol and sufentanil, if necessary, midazolam was added. If no ICP control could be achieved under the aforementioned therapy, sedation was switched to a continuous barbiturate infusion. Sedation was guided by continuous two-channel electroencephalography. Extra ventricular drainage or decompressive hemicraniectomy were discussed on a case-by-case basis.

### Data retrieval

Data were retrieved from the electronic intensive care patient data management system (Intensive Care Manager, V10, Drägerwerk, Lübeck, Germany) with its proprietary data extraction tool (ICMiq V1.4, Drägerwerk, Lübeck, Germany). Additional data were obtained from the electronic patients’ records (Soarian Clinicals 4.5.200, Cerner Health Services, Kansas City, MO, USA) and from the picture archiving and communication system (Centricity, GE Healthcare, Barrington, IL, USA). Oxygen partial pressures (PaO_2_) and time of measurements of all arterial blood gas analyses (ABG) were collected. Furthermore, the following demographic and descriptive data, if applicable, were recorded for each patient: age, sex, weight, height, length of stay in the ICU, duration of ventilation, Charlson Comorbidity Index (CCI), mechanism of injury, initial Glasgow Coma Score (GCS), pupillary reactivity to light, Abbreviated Injury Scale (AIS) for head, neck, face, chest, abdomen, extremities and pelvic girdle, and external injuries, as well as Injury Severity Scale (ISS) [18], Simplified Acute Physiology Score II (SAPS II) [19], conservative and surgical treatment and mortality including the cause of death (i.e. withdrawal of care or brain death). The initial computed tomography (CT) was scanned for the type of intracranial bleeding, signs of herniation and the Rotterdam-CT-Score [20]. Data management was done with Microsoft Excel 2019 (Microsoft Corp., Redmond, WA, USA).

### Oxygen values

**Fig. 1:**
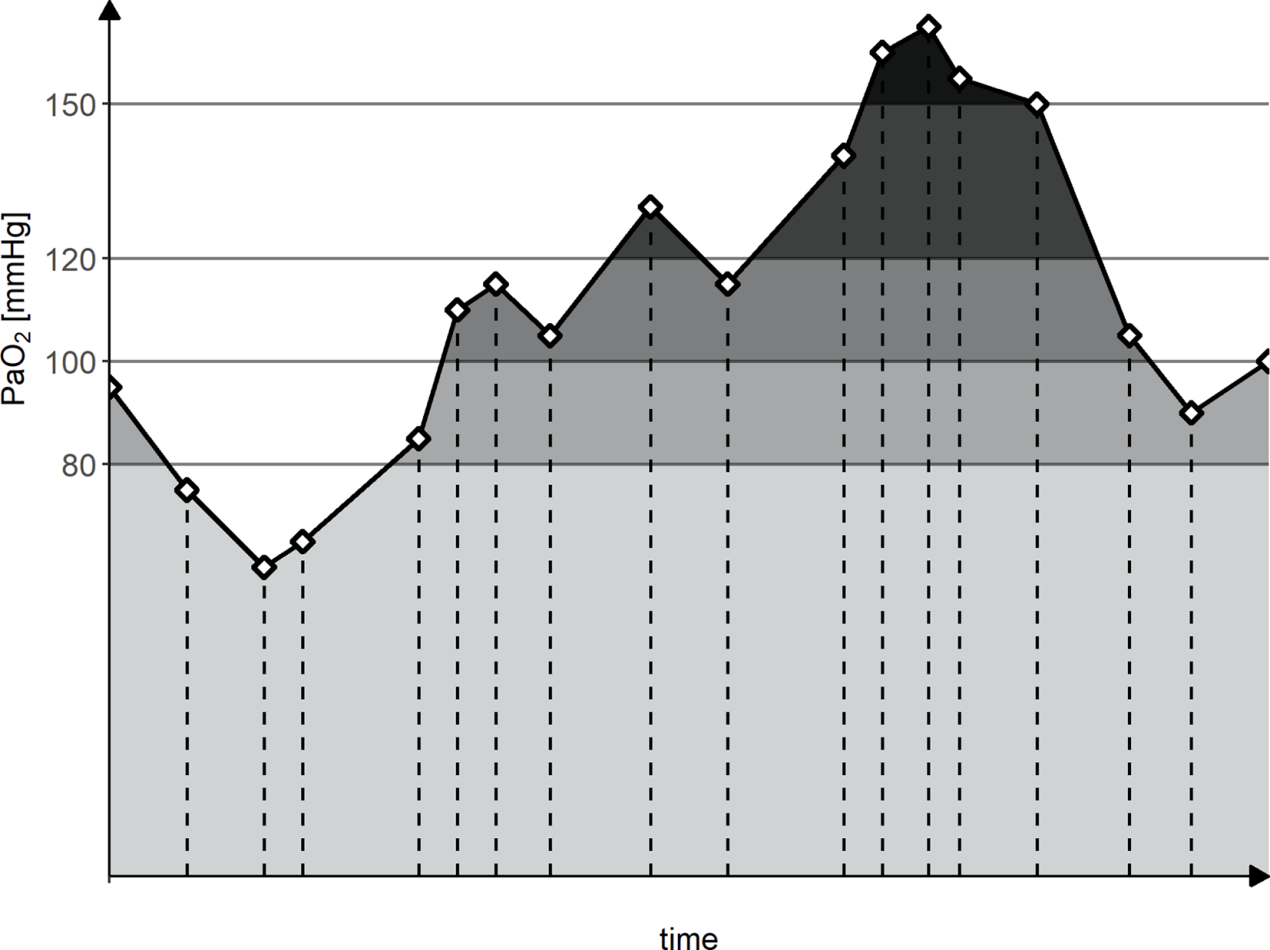
Calculation of integrals. Exemplary calculation of integrals above the defined thresholds. Diamonds and vertical dashed lines represent the obtained arterial blood gas analyses; black line represents the presumed linear change in between; horizontal gray lines depict the thresholds above which the integrals were calculated. mean PaO_2_ = light gray + medium-light gray + medium-dark gray + dark gray + black shaded areas. integral above 80 mmHg = medium-light gray + medium-dark gray + dark gray + black shaded areas. integral above 100 mmHg = medium-dark gray + dark gray + black shaded areas. integral above 120 mmHg = dark gray + black shaded area. integral above 150 mmHg = black shaded area. PaO_2_ = arterial oxygen partial pressure. Modified from Grensemann et al. [17]

Based on the ABGs, which were measured at least every four hours, the following oxygenation parameters were calculated for each patient as described previously [17]: time-weighted mean PaO_2_, taking into account the different time periods between individual measurements, and PaO_2_ integrals above thresholds of 80, 100, 120, and 150 mmHg, using the trapezoidal rule for numerical integration by assuming a linear change between the ABGs (illustrated in Fig. 1). All calculations were performed for four different periods after admission to the ICU: 24 h as hyper-acute phase, 72 h as acute phase, 7 days, and 14 days for long-term oxygen exposure. To ensure comparability, all integrals were calculated as mean integrals per day. For patients who died or were discharged from the ICU before the end of the observation period (supplementary figure S1), calculations were performed using the available data. All calculations of oxygenation parameters were carried out using Visual Basic for Applications (V7.1, Microsoft Corp., Redmond, WA, USA).

### Outcome parameters

The influence of oxygen exposure on in-hospital mortality and functional neurological outcome, assessed by the 5-point Glasgow Outcome Scale (GOS) [21] at discharge and after three to six months, was evaluated. A favorable outcome was defined as GOS 4 or 5, which implies good recovery or independence in activities of daily living [21].

### Statistical analysis

For univariable analyses of patient characteristics and oxygenation parameters, Pearson´s Chi square test for categorial parameters and Student´s t-test for continuous variables were used. To determine the influence of the different oxygenation parameters on the dependent variables in-hospital mortality and favorable outcome at discharge and after three to six months, binary multivariable logistic regression models were generated. Age, CCI, initial GCS, pupillary reactivity to light, AIS for the head, Rotterdam-CT-Score and the SAPS II were selected a priori as covariates for all statistical analyses. To avoid collinearity, the SAPS II was modified and calculated without points for age and GCS. Separate models were calculated for different oxygenation parameters as independent variables and the results were represented as odds ratios (OR) along with the corresponding 95% confidence interval (CI). In addition, we performed two pre-planned sensitivity analyses to verify the statistics by excluding patients who died due to treatment withdrawal or patients with severe chest injury, defined as AIS of 4 or above for the chest. For the determination of optimal PaO_2_ values, we used time-weighted mean PaO_2_ as a continuous parameter and generated logistic regression models using restricted cubic splines (RCS) with 3 knots. Additionally, relative distribution analyses, comparing the distribution of PaO_2_ between patients with and without an advantageous outcome using relative ranks, were used to determine the thresholds of potential optimal oxygen target ranges. All statistical tests were two-tailed and considered to be statistically significant when *p* < 0.05. No data imputation was performed for missing data or loss to follow-up. Statistical analyses were performed using SPSS (version 27, IBM Inc., Armonk, NY, USA), and logistic regression modeling, relative distribution analysis as well as visualization of the data were done using R statistical software (version 4.2.2., R Foundation for Statistical Computing, Vienna, Austria). Data are given as numbers (percentage, %) for categorial parameters, and mean (± standard deviation, SD) or median (interquartile range, IQR) for continuous variables, as applicable.

## Results

**Fig. 2:**
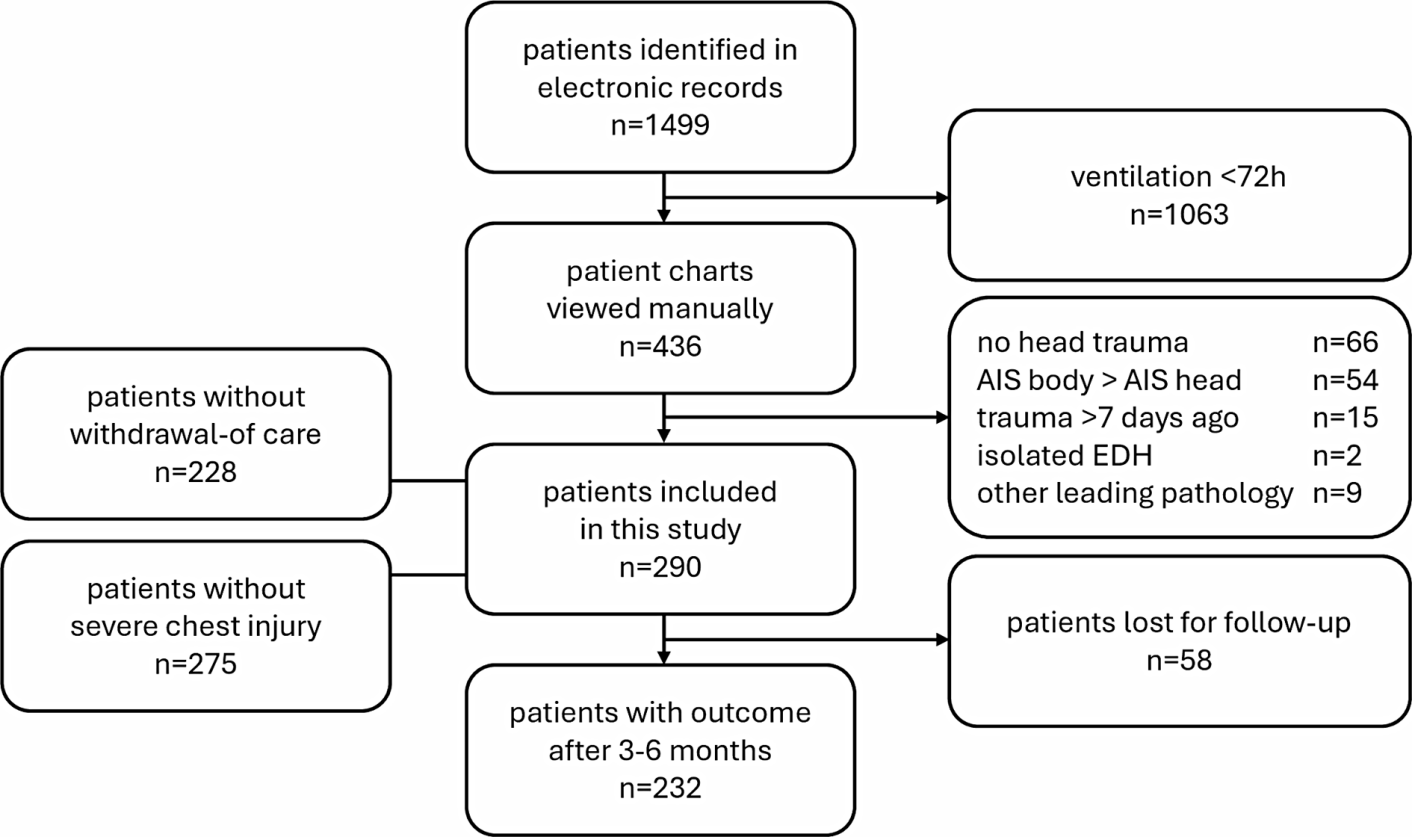
Patient inclusion flowchart. AIS = Abbreviated Injury Scale, EDH = epidural hematoma

From January 2013 to December 2021, 290 TBI patients with 35,810 corresponding ABG measurements fulfilled the inclusion criteria and were included into this study (Fig. 2). The mean patient age was 55.7 (± 21.0) years, and the majority of patients were male (*n* = 219, 75.5%). Table 1 provides a description of patient, trauma and therapy characteristics. In-hospital mortality was 31.7% (*n* = 92), whereby life-sustaining therapy was withdrawn in 62 cases (21.4%) and brain death got ascertained in 27 patients (9.3%). Surviving patients were younger, had fewer previous illnesses and suffered on average less severe injuries (*p* < 0.001). Thirteen patients (4.5%) experienced a favorable neurological outcome at discharge and 72 (24.8%) after three to six months post-injury, though 58 (20%) patients were lost to follow up.

**Table 1:** Patient, trauma and therapy characteristics

Patient characteristics	overall (n=290)	survivors (n=198)	non-survivors (n = 92)
age [years]	55.7 ± 21	51.7 ± 20	64.2 ± 20.6
sex [n]			
female	71 (24.5)	43 (21.7)	28 (30.4)
male	219 (75.5)	155 (78.3)	64 (69.6)
weight [kg]	79.94 ± 12.63	80.76 ± 12.51	78.11 ± 13.02
height [cm]	176.68 ± 8.23	177.60 ± 8.03	174.69 ± 8.37
length of stay in intensive care unit [days]	18.58 ± 12.30	22.62 ± 11.65	9.90 ± 8.64
duration of ventilation [days]	11.48 ± 4.98	12.88 ± 4.42	8.48 ± 4.75
Charlson Comorbidity Index	1.0 [4.0]	1.0 [4.0]	4.0 [4.0]
mechanism of injury			
traffic accident: pedestrian	23 (7.9)	17 (8.6)	6 (6.5)
traffic accident: bicycle	41 (14.1)	30 (15.2)	11 (12.0)
traffic accident: vehicle	32 (11.0)	28 (14.1)	4 (4.3)
traffic accident: motorcycle	22 (7.6)	14 (7.1)	8 (8.7)
low fall (< 3 m)	124 (42.8)	74 (37.4)	50 (54.3)
high fall (> 3 m)	25 (8.6)	18 (9.1)	7 (7.6)
penetrating trauma	3 (1.0)	2 (1.0)	1 (1.1)
other	18 (6.2)	13 (6.5)	5 (5.4)
unknown	7 (2.4)	6 (3.0)	1 (1.1)
Glasgow Coma Score, initial			
13–15	67 (23.1)	46 (23.2)	21 (22.8)
9–12	49 (16.9)	35 (17.7)	14 (15.2)
4–9	78 (26.9)	54 (27.3)	24 (26.1)
3	69 (23.8)	45 (22.7)	24 (26.1)
unknown	27 (9.3)	18 (9.1)	9 (9.8)
pupillary reactivity to light			
both reactive	196 (67.6)	145 (73.2)	51 (55.4)
one reactive	42 (14.5)	27 (13.6)	15 (16.3)
none reactive	42 (14.5)	23 (11.6)	19 (20.7)
unknown	10 (3.4)	3 (1.5)	7 (7.6)
Abbreviated Injury Scale for head			
2	2 (0.7)	1 (0.5)	1 (1.1)
3	39 (13.4)	35 (17.7)	4 (4.3)
4	93 (32.1)	69 (34.8)	24 (26.1)
5	156 (53.8)	93 (47.0)	63 (68.5)
Abbreviated Injury Scale for chest			
0	173 (59.7)	107 (54.0)	66 (71.7)
1–3	102 (35.2)	80 (40.3)	22 (23.9)
4–5	15 (5.1)	11 (5.6)	4 (4.3)
Injury Severity Scale	25.0 [13.0]	25 [15.25]	25 [4.0]
type of intracranial bleeding			
epidural hematoma	22 (7.6)	20 (10.1)	2 (2.2)
subdural hematoma	192 (66.2)	117 (59.1)	75 (81.5)
traumatic subarachnoid hemorrhage	218 (75.2)	151 (76.3)	67 (72.8)
intracerebral hemorrhage	135 (46.6)	95 (48.0)	40 (43.5)
herniation in initial computed tomography	44 (15.2)	13 (6.6)	31 (33.7)
Rotterdam-CT-Score			
1	1 (0.3)	1 (0.5)	0 (0.0)
2	34 (11.7)	28 (14.1)	6 (6.5)
3	132 (45.5)	102 (51.5)	30 (32.6)
4	56 (19.3)	35 (17.7)	21 (22.8)
5	37 (12.8)	14 (7.1)	23 (25.0)
6	16 (5.5)	6 (3.0)	10 (10.9)
unknown	14 (4.8)	12 (6.1)	2 (2.2)
Simplified Acute Physiology Score II	47.5 [21.0]	44.0 [19.5]	53.5 [18.0]
treatment			
conservative	132 (45.5)	94 (47.5)	38 (41.3)
external ventricle drainage	56 (19.3)	39 (19.7)	17 (18.5)
decompressive hemicraniectomy	73 (25.2)	49 (24.7)	24 (26.1)
surgical care of epidural hematoma	18 (6.2)	14 (7.1)	4 (4.3)
surgical care of subdural hematoma	87 (30.0)	50 (25.3)	37 (40.2)
surgical care of intracerebral hemorrhage	18 (6.2)	12 (6.1)	6 (6.5)
other	9 (3.1)	6 (3.0)	3 (3.3)
mortality	92 (31.7)		
brain death	27 (9.3)		
withdrawal of care	62 (21.4)		

Data are given as numbers and percentage in parentheses, mean ± standard deviation or median and interquartile range in brackets, as applicable

The time-weighted mean PaO_2_ were 98 (± 23), 91 (± 14), 90 (± 24) and 89 (± 24) mmHg for the observational durations of 1, 3, 7 and 14 days, respectively. Hypoxia, defined as a time-weighted mean PaO_2_ below 60 mmHg, was evident only for the 1- and 7-day time period, only affecting one patient each.

On univariable analyses of mean PaO_2_ and integrals above the set thresholds, a significant association of hyperoxia within the first 7 and 14 days after admission with increased in-hospital mortality (supplementary table S1) and impaired neurological outcome in the follow-up after three to six months (supplementary table S2) could be demonstrated. Regarding the functional outcome at patient discharge, only 13 patients achieved a favorable outcome at this time and hyperoxia could not be determined as a predicting factor for improved or compromised GOS (supplementary table S3). Sensitivity analyses excluding patients with concomitant severe chest injury or with withdrawal of life-support are given in the supplement (supplementary tables S4– S7).

**Fig. 3:**
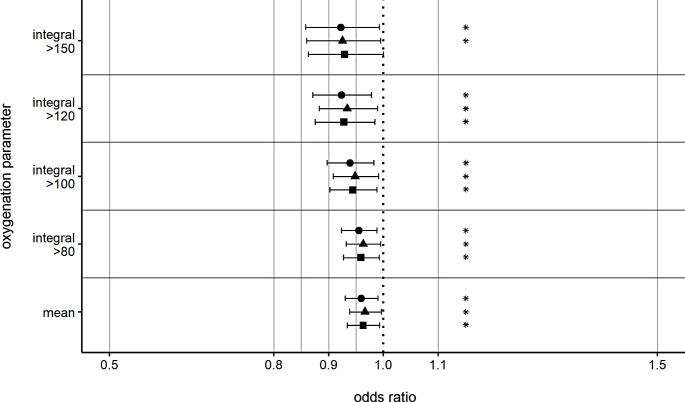
Odds ratios for favorable GOS after 3–6 months for hyperoxia over 24 h. Odds ratios for favorable Glasgow Outcome Score (≥ 4) for the integrals depicting a mean increase of 1 mmHg/day for 24 h above the respective threshold. Separate models were calculated for each oxygenation parameter. ⬤ = statistical analysis with the overall population. ▲= sensitivity analysis without patients with withdrawal-of-care. ■ = sensitivity analysis without patients with severe chest injury. Integral = arterial oxygen partial pressure integral above respective threshold in mmHg. Mean = mean arterial oxygen partial pressure in mmHg. * *p* < 0.05 versus baseline. Error bars present the 95% confidence intervals

**Fig. 4:**
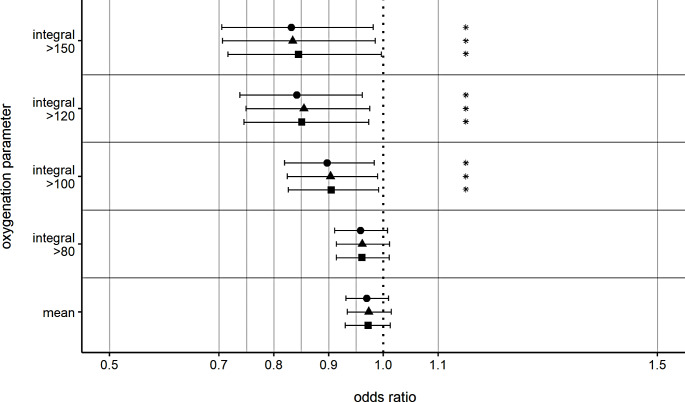
Odds ratios for favorable GOS after 3–6 months for hyperoxia over 72 h. Odds ratios for favorable Glasgow Outcome Score (≥ 4) for the integrals depicting a mean increase of 1 mmHg/day for 72 h above the respective threshold. Separate models were calculated for each oxygenation parameter. ⬤ = statistical analysis with the overall population. ▲= sensitivity analysis without patients with withdrawal-of-care. ■ = sensitivity analysis without patients with severe chest injury. Integral = arterial oxygen partial pressure integral above respective threshold in mmHg. Mean = mean arterial oxygen partial pressure in mmHg. * *p* < 0.05 versus baseline. Error bars present 95% confidence intervals

For in-hospital mortality, no significant association of time-weighted mean PaO_2_ or PaO_2_ integrals could be shown in the multivariable analyses, although there was a trend toward higher in-hospital mortality (supplementary table S8). Concerning favorable outcomes at three to six months, the multivariable binary logistic regression analyses showed a significant influence of time-weighted mean PaO_2_ (OR 0.959, 95%CI 0.930–0.990, *p* = 0.009) and all calculated integrals above the thresholds of 80 mmHg (OR 0.955, 95%CI 0.923–0.988, *p* = 0.008), 100 mmHg (OR 0.939, 95%CI 0.897–0.982, *p* = 0.006), 120 mmHg (OR 0.923, 95%CI 0.871–0.978, *p* = 0.007) and 150 mmHg (OR 0.922, 95%CI 0.858–0.992, *p* = 0.029) for the first 24 h after admission (Fig. 3, supplementary table S9). A favorable outcome was more likely, when time-weighted mean PaO_2_ and PaO_2_-integrals above the thresholds were lower. Similar logistic regression analyses for the first 72 h after admission revealed a statistically significant poorer neurological outcome after three to six months for higher integrals above the thresholds of 100 mmHg (OR 0.897, 95%CI 0.819–0.983, *p* = 0.020), 120 mmHg (OR 0.842, 95%CI 0.738–0.961, *p* = 0.011) and 150 mmHg (OR 0.832, 95%CI 0.705–0.981, *p* = 0.029) (Fig. 4, supplementary table S9). The multivariable logistic regression sensitivity analyses are shown in Figs. 3 and 4, and the supplementary tables S10 and S11.

**Fig. 5:**
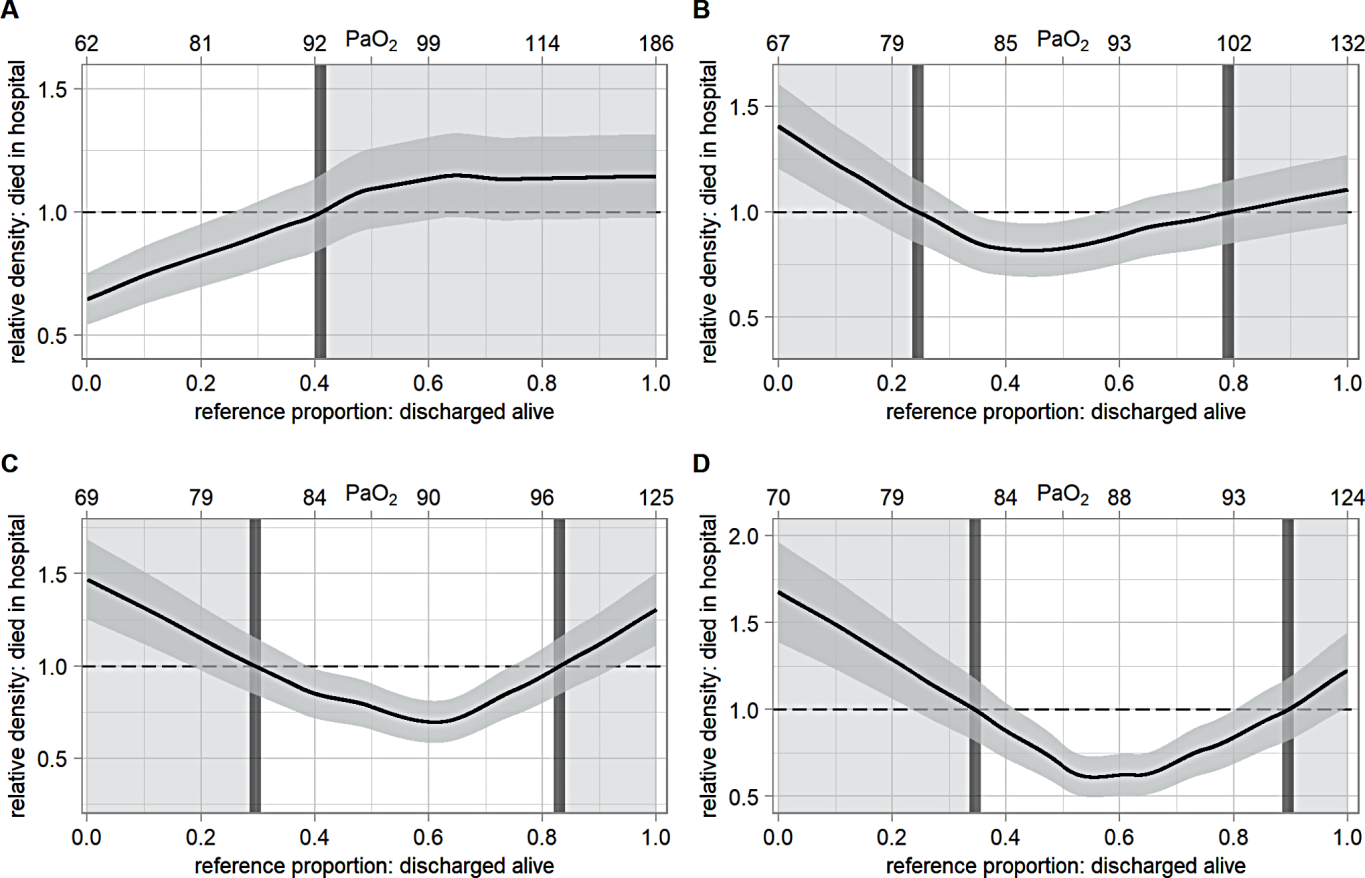
Relative distribution analysis for in-hospital mortality, multivariable adjusted. Relative distribution analysis for in-hospital mortality, adjusted for: age, Charlson Comorbidity Index, initial Glasgow Coma Score, pupillary reactivity to light, Rotterdam-CT-Score, Abbreviated Injury Scale for head and Simplified Acute Physiology Score II without points for age and initial Glasgow Coma Score. Upper x-axis displays the time-weighted mean PaO_2_ corresponding to the reference proportion, rounded to the nearest natural number. Middle gray area depicts the 95%CI. The white background area separated by the dark gray bars represents the optimal oxygen target range revealed by the relative distribution analysis, the lighter gray background area presents the range beyond. PaO_2_ = arterial partial pressure of oxygen. 95%CI = 95% confidence interval. A = on day 1.PaO_2_ of lower threshold: not applicable. PaO_2_ of upper threshold: 92 mmHg = OR 1.00, 95%CI = 0.85–1.15. B = admission to day 3. PaO_2_ of lower threshold: 80 mmHg = OR 1.00, 95%CI = 0.85–1.15. PaO_2_ of upper threshold: 101 mmHg = OR 1.00, 95%CI = 0.85–1.15.  C = admission to day 7. PaO_2_ of lower threshold: 81 mmHg = OR 1.00, 95%CI = 0.85–1.15. PaO_2_ of upper threshold: 97 mmHg = OR 1.00, 95%CI = 0.85–1.16. D = admission to day 14. PaO_2_ of lower threshold: 83 mmHg = OR 1.00, 95%CI = 0.82–1.18. PaO_2_ of upper threshold: 96 mmHg = OR 1.00, 95%CI = 0.82–1.18

**Fig. 6:**
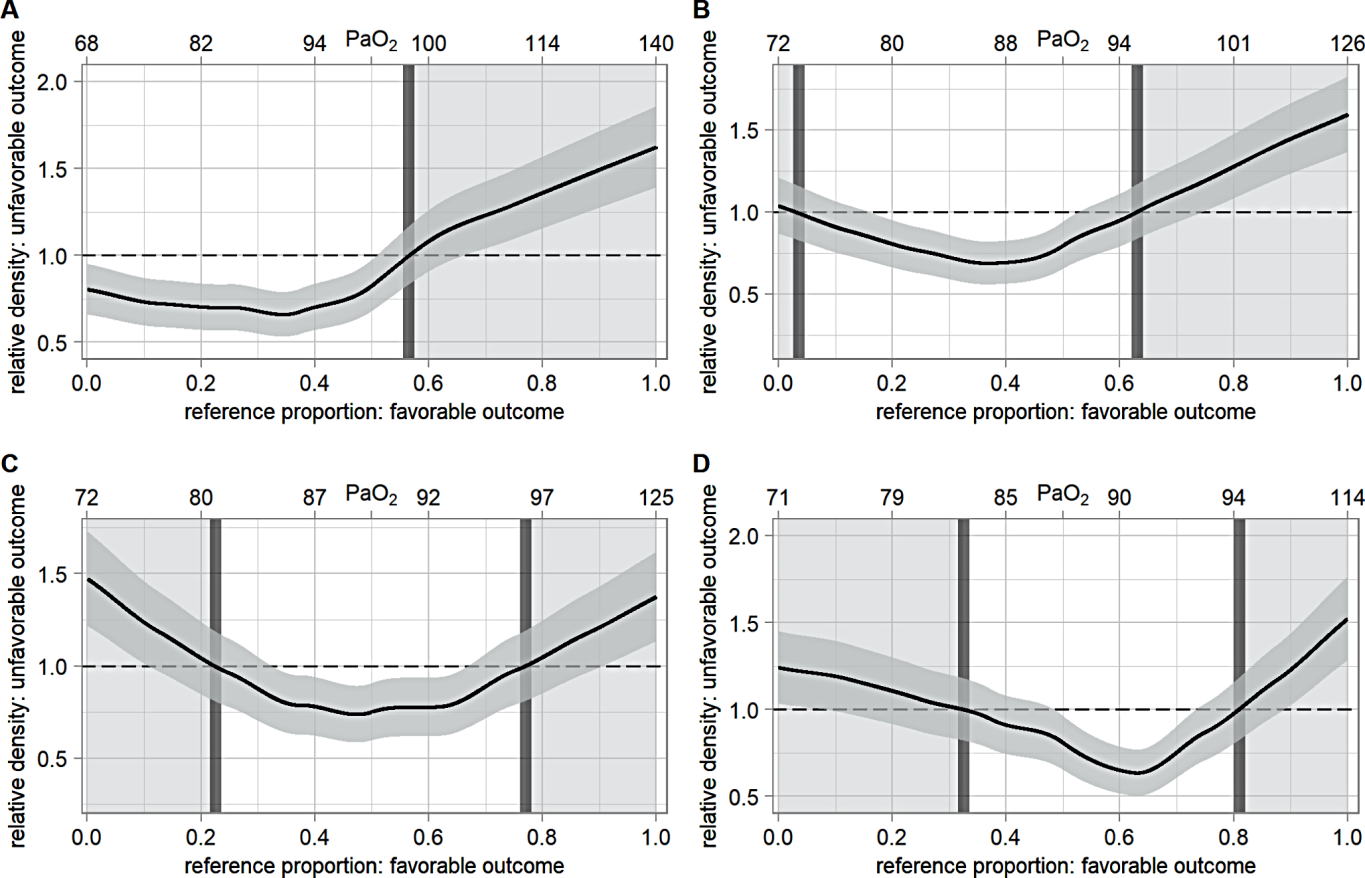
Relative distribution analysis for unfavorable GOS after 3–6 months, multivariable adjusted. Relative distribution analysis for unfavorable GOS after 3–6 months, adjusted for: age, Charlson Comorbidity Index, initial Glasgow Coma Score, pupillary reactivity to light, Rotterdam-CT-Score, Abbreviated Injury Scale for head and Simplified Acute Physiology Score II without points for age and initial Glasgow Coma Score. Upper x-axis displays the time-weighted mean PaO_2_ corresponding to the reference proportion, rounded to the nearest natural number. Middle gray area depicts the 95%CI. The white background area separated by the dark gray bars represents the optimal oxygen target range revealed by the relative distribution analysis, the lighter gray background area presents the range beyond. PaO_2_ = arterial partial pressure of oxygen. 95%CI = 95% confidence interval. A = on day 1. PaO_2_ of lower threshold: not applicable. PaO_2_ of upper threshold: 99 mmHg = OR 1.00, 95%CI = 0.83–1.17. B = admission to day 3. PaO_2_ of lower threshold: 75 mmHg = OR 1.00, 95%CI = 0.83–1.17. PaO_2_ of upper threshold: 96 mmHg = OR 1.00, 95%CI = 0.83–1.17.  C = admission to day 7. PaO_2_ of lower threshold: 80 mmHg = OR 1.00, 95%CI = 0.81–1.19. PaO_2_ of upper threshold: 97 mmHg = OR 1.00, 95%CI = 0.81–1.19. D = admission to day 14. PaO_2_ of lower threshold: 83 mmHg = OR 1.00, 95%CI = 0.82–1.18. PaO_2_ of upper threshold: 94 mmHg = OR 1.00, 95%CI = 0.82–1.18

Modeling time-weighted mean PaO_2_ as a continuous parameter in logistic regression models with restricted cubic splines, a U-shaped relationship between PaO_2_ and in-hospital mortality as well as neurological outcome at three to six months was observed, but did not reach statistical significance, neither in the univariate or multivariable analyses (supplement figures S2– S5). For in-hospital mortality, after multivariable adjustment, the optimal PaO_2_ was 88 mmHg for the first 3, 7 and 14 days (supplementary figure S4). Regarding the GOS after three to six months, the most favorable PaO_2_ was 88 mmHg for mean PaO_2_ after 7 and 14 days in the multivariable model (supplementary figure S5). In the logistic regression models not depicting a U-shape (supplementary figures S2 and S5), odds ratios were adjusted to an arbitrarily chosen PaO_2_-value. The relative distribution analyses for in-hospital mortality, adjusted for the same covariates as before, revealed 92 mmHg as potential upper threshold on day 1 and 80–101 mmHg, 81–97 mmHg and 83–96 mmHg as potential oxygen target ranges for mean PaO_2_ in TBI patients for the first 3, 7 and 14 days, respectively (Fig. 5). Concerning GOS after 3 to 6 months, the multivariable adjusted relative distribution analyses identified 99 mmHg as potential upper threshold on day 1 and 75–96 mmHg, 80–97 mmHg and 83–94 mmHg as potential thresholds for the first 3, 7 and 14 days (Fig. 6).

Additional details concerning the distribution of time-weighted mean PaO_2_ (supplementary figure S6), the discontinuation of mechanical ventilation during the first 14 days after admission (supplementary table S12, supplementary figure S1), a comparison of time-weighted mean PaO_2_ before and after extubation (supplementary table S13) and the cause of in-hospital mortality grouped after time-weighted mean PaO_2_ (supplementary table S14 and S15) are provided in the supplement.

## Discussion

In this cohort of 290 patients with TBI ventilated for at least 72 h, hyperoxia during the first three days of ICU stay was significantly associated with an unfavorable neurological outcome three to six months post-injury.

Maintaining adequate tissue oxygenation to avoid brain tissue hypoxia while minimizing the risk of complications associated with excessive oxygen exposure is paramount to optimize outcomes for neurocritical care patients receiving oxygen therapy. Above a PaO_2_ of 80 mmHg, corresponding to an oxygen saturation (SpO_2_) of approximately 96%, hemoglobin physiologically binds and transports only a negligible amount of additional oxygen due to its sigmoidal binding curve [2]. Beyond this oxygen partial pressure, further oxygen supplementation may not enhance cerebral oxygenation and therefore metabolism [22], but negative side-effects associated with hyperoxia may become more prevalent and outweigh the benefit of the small amount of additional oxygen delivery. Due to this underlying physiology, this study examined lower thresholds than previous studies [8, 9, 12, 14, 15, 23–25] and was able to demonstrate a significant impact of hyperoxia on clinical outcome of TBI patients, favoring a lower range of oxygen partial pressure. Nevertheless, this range aligns with current German guidelines, which recommend a target range for oxygen saturation of 92–96% in the acute care of adult patient [26], 90–94% for mechanically ventilated patients [27] and ≥ 90% in TBI patients [4] as well as patients with severe/multiple injuries [28], while no recommendation on oxygen targets is given in the current guideline of the Brain Trauma Foundation [29]. However, simultaneous monitoring of PaO_2_ and brain tissue oxygenation (PbtO_2_) revealed that the PaO_2_ required to maintain a PbtO_2_ above the critical threshold for tissue hypoxia of 20 mmHg is found at 94 mmHg [30], which is in line with the oxygen target range associated with the most favorable outcomes in this study.

Accessing oxygen exposure using integral calculation above multiple thresholds, integrals above higher thresholds showed a stronger influence with lower odds ratios for a favorable neurological outcome at three to six months, indicating dose-dependent adverse effects of hyperoxia within the first three days after trauma. With longer observation periods of seven and fourteen days, the results showed a loss of statistical significance, but a discernible trend persisted and still supports the approach of lower oxygen targets. In contrast, in our previous study with similar statistical analyses on patients with aneurysmal subarachnoid hemorrhage the results became increasingly significant over time and reached statistical significance after 14 days [17], which we interpreted as a sign of time-dependent accumulation of cell injury through oxygen toxicity. The effects of supranormal PaO_2_ values and therefore optimal oxygen target values and ranges may therefore differ between specific populations of patients depending on the underlying pathology with individual requirements and hazards regarding oxygen exposure [31]. Future studies need to address the possibility of changing oxygenation targets over the course of therapy. While in our study of TBI patients an overall target PaO_2_ of approximately 80 to 100 mmHg was associated with the most favorable outcomes, it is important to note that the oxygen integrals above the lower thresholds also include the integrals above the higher thresholds, and therefore the optimal PaO_2_ revealed by the logistic regression models or optimal target range identified in the relative distribution analyses may be within or above the lower integral thresholds.

Liberal oxygen supplementation promotes formation of reactive oxygen species (ROS). Excessive production of ROS can exceed the capacity of antioxidant defense mechanisms leading to a time- and dose-dependent accumulation of ROS and thus oxygen toxicity. This may induce oxidative stress damaging cellular components, including cell membrane, lipids, proteins, signaling pathways and DNA, promoting cellular dysfunction and tissue damage [3, 32]. In addition, hyperoxia can cause an inflammatory response along with a release of pro-inflammatory cytokines, such as interleukin 6 (IL-6), and an activation of immune cells, triggering neuroinflammation [33] and pulmonary diseases such as pneumonia [34]. Furthermore, hyperoxia can disrupt perfusion through the induction of vasoconstriction, particularly in cerebral vessels [35], decreasing oxygen delivery to the brain, and potentially diminishing the cerebral autoregulation [36], even with improved cerebral regional oxygen saturation [37]. Additional mechanisms contributing to oxygen toxicity have been identified, including breakdown of the blood-brain barrier, and direct mitochondrial damage [38, 39]. Despite this underlying pathophysiology, Lang et al. could not demonstrate a significant increase of ROS, IL-6 or neuron-specific enolase (NSE), as marker for neurological cell damage in TBI patients ventilated with a higher fraction of inspired oxygen (FiO_2_) [16].

Presently, no prospective studies have assessed long-term oxygenation targets regarding clinical outcome parameters in patients suffering from TBI, and retrospective studies yielded inconsistent results. Two prospective interventional studies examined the influence of short-term hyperoxia, postulating beneficial effects of hyperoxia due to improved brain metabolism [40, 41]. Retrospective studies observed higher PaO_2_ values in surviving patients [10]. Potential therapeutic benefits from hyperoxia above 100 mmHg [24], 150 mmHg [42], and 400 mmHg were observed in other studies [15], presumably by mitigating secondary brain injury by effectively preventing hypoxia. On the other hand, hyperoxia has been linked to adverse outcomes in TBI patients [11, 43, 44] and the majority of studies have shown a U-shaped relationship between PaO_2_ levels and clinical outcome, with both hypoxia and hyperoxia being linked to deteriorating patient prognosis [7–9, 13, 14, 23]. The resulting optimal oxygen target ranges varied between a PaO_2_ of 75–100 mmHg [13], 100–200 mmHg [8], 150–200 mmHg [7], 60–300 mmHg [14] and 110–487 mmHg [9] and a SpO_2_ of 95–98% [45]. The results of our study align with the general conclusion of targeting normoxia and avoiding hypoxia as well as hyperoxia, indicating a lower oxygen target range around approximately 80 to 100 mmHg.

Our study has certain limitations. The retrospective and exploratory design of the study allowed us to demonstrate an association between oxygen exposure and clinical outcome in patients with TBI, even though this does not necessarily translate into a causal relationship, and despite comprehensive adjustment other influencing factors on the clinical outcome cannot be excluded. Therefore randomized controlled trials investigating the impact of different oxygenation targets on the outcomes of ventilated patients with TBI would be desirable. To limit confounding, we excluded patients with less than 72 h of mechanical ventilation and thus an early fatal outcome, as well as patients with multiple trauma in whom the other injuries were more severe than the TBI. The calculation of time-weighted mean and oxygen integrals from numerous ABGs during the ICU stay enabled us to explore the long-term oxygenation without recording the oxygenation situation directly in the brain tissue performing PbtO_2_-measurement. However, it remains possible that hypoxic phases, e.g. during acute ventilation problems, or hyperoxic phases, e.g. during preoxygenation before interventions, occurred in the meantime. Our data set is too small to address additional questions, e.g., whether the optimal oxygen target range changes over time after acute brain injury, which needs to be addressed by future research. In addition, other factors that may influence individual oxygen requirements, such as patient age, certain chronic diseases, or injury severity, should be investigated.

## Conclusion

In this study, investigating the influence of oxygen exposure in critically ill patients with TBI, hyperoxia during the first three days of ICU treatment was associated with a reduced favorable functional outcome after three to six months. Additionally, we could demonstrate a U-shaped relationship between oxygen exposure and clinical outcome, favoring a target range from approximately 80 to 100 mmHg. These results support the approach of a conservative and ABG guided administration of oxygen in TBI patients and emphasize the need for further prospective research into the effects of long-term hyperoxia and the need to evaluate an optimal target range of oxygen supply for critically ill TBI patients to improve patient care.

## Electronic supplementary material

Below is the link to the electronic supplementary material.

Supplementary Material 1

## Data Availability

The dataset used during this study is available from the corresponding author upon reasonable request.

## References

[1] Wu H, Gong L, Gu JC, Xing HW, Qian ZX, Mao Q. Proper partial pressure of arterial oxygen for patients with traumatic brain Injury. Med Sci Monit. 2021;27:e932318. 10.12659/msm.932318. 34663780 10.12659/MSM.932318 PMC8540035

[2] Grensemann J, Wachs C, Kluge S. Oxygen therapy in emergency and intensive care medicine. Dtsch Med Wochenschr. 2021;146(2):108–. 10.1055/a-0948-8363. 20. 33465807 10.1055/a-0948-8363

[3] Helmerhorst HJ, Schultz MJ, van der Voort PH, de Jonge E, van Westerloo DJ. Bench-to-bedside review: the effects of hyperoxia during critical illness. Crit Care. 2015;19(1):284. 10.1186/s13054-015-0996-4. 26278383 10.1186/s13054-015-0996-4 PMC4538738

[4] Firsching R, Rickels E, Mauer UM, Sakowitz OW, Messing-Jünger M, Engelhard K et al. German S2e Guideline: Traumatic Brain Injury in adults 2015. [Available from: https://register.awmf.org/assets/guidelines/008-001l_S2e_Schaedelhirntrauma_SHT_Erwachsene_2015-12-abgelaufen.pdf 10.1055/s-0037-1599239 28482371

[5] Hirunpattarasilp C, Shiina H, Na-Ek N, Attwell D. The Effect of Hyperoxemia on neurological outcomes of adult patients: a systematic review and Meta-analysis. Neurocrit Care. 2022. 10.1007/s12028-021-01423-w. 35099713 10.1007/s12028-021-01423-w PMC9110471

[6] Girardis M, Busani S, Damiani E, Donati A, Rinaldi L, Marudi A, et al. Effect of conservative vs conventional oxygen therapy on Mortality among patients in an intensive care unit: the Oxygen-ICU Randomized Clinical Trial. JAMA. 2016;316(15):1583–9. 10.1001/jama.2016.11993. 27706466 10.1001/jama.2016.11993

[7] Alali AS, Temkin N, Vavilala MS, Lele AV, Barber J, Dikmen S, et al. Matching early arterial oxygenation to long-term outcome in severe traumatic brain injury: target values. J Neurosurg. 2019;132(2):537–44. 10.3171/2018.10.Jns18964. 30738409 10.3171/2018.10.JNS18964

[8] Brenner M, Stein D, Hu P, Kufera J, Wooford M, Scalea T. Association between early hyperoxia and worse outcomes after traumatic brain injury. Arch Surg. 2012;147(11):1042–6. 10.1001/archsurg.2012.1560. 22801994 10.1001/archsurg.2012.1560

[9] Davis DP, Meade W, Sise MJ, Kennedy F, Simon F, Tominaga G, et al. Both hypoxemia and extreme hyperoxemia may be detrimental in patients with severe traumatic brain injury. J Neurotrauma. 2009;26(12):2217–23. 10.1089/neu.2009.0940. 19811093 10.1089/neu.2009.0940

[10] Fujita M, Oda Y, Yamashita S, Kaneda K, Kaneko T, Suehiro E, et al. Early-Stage Hyperoxia is Associated with favorable neurological outcomes and survival after severe traumatic brain Injury: a post-hoc analysis of the Brain Hypothermia Study. J Neurotrauma. 2017;34(8):1565–70. 10.1089/neu.2016.4753. 27958774 10.1089/neu.2016.4753

[11] Kılınç Z, Ayyıldız EA, Kaya E, Sahin AS. The Effect of Oxygenation on Mortality in patients with Head Injury. Cureus. 2023;15(1):e34385. 10.7759/cureus.34385. 36874741 10.7759/cureus.34385 PMC9976649

[12] Ó Briain D, Nickson C, Pilcher DV, Udy AA. Early Hyperoxia in patients with traumatic brain Injury admitted to Intensive Care in Australia and New Zealand: a retrospective Multicenter Cohort Study. Neurocrit Care. 2018;29(3):443–. 10.1007/s12028-018-0553-5. 51. 29949002 10.1007/s12028-018-0553-5

[13] Raj R, Bendel S, Reinikainen M, Kivisaari R, Siironen J, Lång M, et al. Hyperoxemia and long-term outcome after traumatic brain injury. Crit Care. 2013;17(4):R177. 10.1186/cc12856. 23958227 10.1186/cc12856 PMC4056982

[14] Rincon F, Kang J, Vibbert M, Urtecho J, Athar MK, Jallo J. Significance of arterial hyperoxia and relationship with case fatality in traumatic brain injury: a multicentre cohort study. J Neurol Neurosurg Psychiatry. 2014;85(7):799–805. 10.1136/jnnp-2013-305505. 23794718 10.1136/jnnp-2013-305505

[15] Davis DP, McKnight B, Meier E, Drennan IR, Newgard C, Wang HE, et al. Higher oxygenation is Associated with Improved Survival in severe traumatic brain Injury but not traumatic shock. Neurotrauma Rep. 2023;4(1):51–63. 10.1089/neur.2022.0065. 36726869 10.1089/neur.2022.0065 PMC9886195

[16] Lång M, Skrifvars MB, Siironen J, Tanskanen P, Ala-Peijari M, Koivisto T, et al. A pilot study of hyperoxemia on neurological injury, inflammation and oxidative stress. Acta Anaesthesiol Scand. 2018;62(6):801–10. 10.1111/aas.13093. 29464691 10.1111/aas.13093

[17] Grensemann J, Mader MM, Westphal M, Kluge S, Czorlich P. Hyperoxia is dose-Dependently Associated with an increase of unfavorable outcomes in ventilated patients with Aneurysmal Subarachnoid Hemorrhage: a retrospective cohort study. Neurocrit Care. 2022;37(2):523–. 10.1007/s12028-022-01534-y. 30. 35672497 10.1007/s12028-022-01534-y PMC9519732

[18] Baker SP, O’Neill B, Haddon W Jr., Long WB. The injury severity score: a method for describing patients with multiple injuries and evaluating emergency care. J Trauma. 1974;14(3):187–96. 4814394

[19] Le Gall JR, Lemeshow S, Saulnier F. A new simplified Acute Physiology score (SAPS II) based on a European/North American multicenter study. JAMA. 1993;270(24):2957–63. 10.1001/jama.270.24.2957. 8254858 10.1001/jama.270.24.2957

[20] Maas AI, Hukkelhoven CW, Marshall LF, Steyerberg EW. Prediction of outcome in traumatic brain injury with computed tomographic characteristics: a comparison between the computed tomographic classification and combinations of computed tomographic predictors. Neurosurgery. 2005;57(6):1173–82. 10.1227/01.neu.0000186013.63046.6b. discussion– 82. 16331165 10.1227/01.neu.0000186013.63046.6b

[21] Jennett B, Bond M. Assessment of outcome after severe brain damage. Lancet. 1975;1(7905):480–4. 10.1016/s0140-6736(75)92830-5. 46957 10.1016/s0140-6736(75)92830-5

[22] Magnoni S, Ghisoni L, Locatelli M, Caimi M, Colombo A, Valeriani V, et al. Lack of improvement in cerebral metabolism after hyperoxia in severe head injury: a microdialysis study. J Neurosurg. 2003;98(5):952–8. 10.3171/jns.2003.98.5.0952. 12744353 10.3171/jns.2003.98.5.0952

[23] Asher SR, Curry P, Sharma D, Wang J, O’Keefe GE, Daniel-Johnson J, et al. Survival advantage and PaO2 threshold in severe traumatic brain injury. J Neurosurg Anesthesiol. 2013;25(2):168–. 10.1097/ANA.0b013e318283d350. 73. 23343758 10.1097/ANA.0b013e318283d350

[24] Vrettou CS, Giannakoulis VG, Gallos P, Kotanidou A, Siempos II. Effect of different early oxygenation levels on clinical outcomes of patients presenting in the Emergency Department with severe traumatic brain Injury. Ann Emerg Med. 2023;81(3):273–. 10.1016/j.annemergmed.2022.09.026. 81. 36402630 10.1016/j.annemergmed.2022.09.026

[25] Weeden M, Bailey M, Gabbe B, Pilcher D, Bellomo R, Udy A. Functional outcomes in patients admitted to the Intensive Care Unit with Traumatic Brain Injury and exposed to Hyperoxia: a retrospective Multicentre Cohort Study. Neurocrit Care. 2021;34(2):441–8. 10.1007/s12028-020-01033-y. 32632905 10.1007/s12028-020-01033-y PMC7338132

[26] Gottlieb J, Capetian P, Hamsen U, Janssens U, Karagiannidis C, Kluge S, et al. German S3 Guideline: Oxygen Therapy in the Acute Care of adult patients. Respiration. 2022;101(2):214–52. 10.1159/000520294. 34933311 10.1159/000520294

[27] Fichtner F, Moerer O, Weber-Carstens S, Nothacker M, Kaisers U, Laudi S. Clinical Guideline for treating Acute respiratory insufficiency with invasive ventilation and extracorporeal membrane oxygenation: evidence-based recommendations for choosing modes and setting parameters of mechanical ventilation. Respiration. 2019;98(4):357–. 10.1159/000502157. 72. 31505511 10.1159/000502157

[28] Deutsche Gesellschaft für Unfallchirurgie e.V. S3-Leitlinie Polytrauma/Schwerverletzen-Behandlung (AWMF Registernummer 187– 023), Version 4.0 (31.12.2022), available under https://register.awmf.org/de/leitlinien/detail/187-023

[29] Carney N, Totten AM, O’Reilly C, Ullman JS, Hawryluk GWJ, Bell MJ et al. Guidelines for the management of severe traumatic brain Injury, Fourth Edition. Neurosurgery. 2017;80(1). 10.1227/NEU.0000000000001432 27654000

[30] Dellazizzo L, Demers SP, Charbonney E, Williams V, Serri K, Albert M, et al. Minimal PaO2 threshold after traumatic brain injury and clinical utility of a novel brain oxygenation ratio. J Neurosurg. 2018;1–9. 10.3171/2018.5.Jns18651. 10.3171/2018.5.JNS18651 30485198

[31] Singer M, Young PJ, Laffey JG, Asfar P, Taccone FS, Skrifvars MB, et al. Dangers of hyperoxia. Crit Care. 2021;25(1):440. 10.1186/s13054-021-03815-y. 34924022 10.1186/s13054-021-03815-y PMC8686263

[32] Ottolenghi S, Sabbatini G, Brizzolari A, Samaja M, Chiumello D. Hyperoxia and oxidative stress in anesthesia and critical care medicine. Minerva Anestesiol. 2020;86(1):64–75. 10.23736/s0375-9393.19.13906-5. 31680497 10.23736/S0375-9393.19.13906-5

[33] Blasiole B, Bayr H, Vagni VA, Janesko-Feldman K, Cheikhi A, Wisniewski SR, et al. Effect of hyperoxia on resuscitation of experimental combined traumatic brain injury and hemorrhagic shock in mice. Anesthesiology. 2013;118(3):649–. 10.1097/ALN.0b013e318280a42d. 63. 23299361 10.1097/ALN.0b013e318280a42d

[34] Staehr-Rye AK, Meyhoff CS, Scheffenbichler FT, Vidal Melo MF, Gätke MR, Walsh JL, et al. High intraoperative inspiratory oxygen fraction and risk of major respiratory complications. Br J Anaesth. 2017;119(1):140–9. 10.1093/bja/aex128.28974067 10.1093/bja/aex128

[35] Bulte DP, Chiarelli PA, Wise RG, Jezzard P. Cerebral perfusion response to hyperoxia. J Cereb Blood Flow Metab. 2007;27(1):69–75. 10.1038/sj.jcbfm.9600319. 16670698 10.1038/sj.jcbfm.9600319

[36] Sahoo S, Sheshadri V, Sriganesh K, Madhsudana Reddy KR, Radhakrishnan M, Umamaheswara Rao GS. Effect of Hyperoxia on Cerebral Blood Flow Velocity and Regional Oxygen Saturation in patients operated on for severe traumatic Brain Injury-the influence of cerebral blood Flow Autoregulation. World Neurosurg. 2017;98:211–6. 10.1016/j.wneu.2016.10.116. 27810458 10.1016/j.wneu.2016.10.116

[37] Ciliberti P, Cardim D, Giardina A, Groznik M, Ball L, Giovannini M, et al. Effects of short-term hyperoxemia on cerebral autoregulation and tissue oxygenation in acute brain injured patients. Front Physiol. 2023;14:1113386. 10.3389/fphys.2023.1113386. 36846344 10.3389/fphys.2023.1113386 PMC9944047

[38] Busani S, Sarti M, Serra F, Gelmini R, Venturelli S, Munari E, et al. Revisited Hyperoxia Pathophysiology in the Perioperative setting: a narrative review. Front Med (Lausanne). 2021;8:689450. 10.3389/fmed.2021.689450. 34746165 10.3389/fmed.2021.689450 PMC8569225

[39] Stuby J, Kaserer A, Ott S, Ruetzler K, Rössler J. Perioperative hyperoxia-more harmful than beneficial? Anaesthesiologie. 2023;72(5):342–7. 10.1007/s00101-023-01274-4. 37084143 10.1007/s00101-023-01274-4

[40] Taher A, Pilehvari Z, Poorolajal J, Aghajanloo M. Effects of Normobaric Hyperoxia in Traumatic Brain Injury: a Randomized Controlled Clinical Trial. Trauma Mon. 2016;21(1):e26772. 10.5812/traumamon.26772. 27218057 10.5812/traumamon.26772 PMC4869427

[41] Tolias CM, Reinert M, Seiler R, Gilman C, Scharf A, Bullock MR. Normobaric hyperoxia–induced improvement in cerebral metabolism and reduction in intracranial pressure in patients with severe head injury: a prospective historical cohort-matched study. J Neurosurg. 2004;101(3):435–44. 10.3171/jns.2004.101.3.0435. 15352601 10.3171/jns.2004.101.3.0435

[42] Wiginton Jt, Brazdzionis J, Patchana T, Dorkoski R, Miulli DE, Sweiss R, et al. Optimal partial pressure of Oxygen affects outcomes in patients with severe traumatic brain Injury. Cureus. 2020;12(8):e9964. 10.7759/cureus.9964. 32983668 10.7759/cureus.9964 PMC7510506

[43] Rezoagli E, Petrosino M, Rebora P, Menon DK, Mondello S, Cooper DJ, et al. High arterial oxygen levels and supplemental oxygen administration in traumatic brain injury: insights from CENTER-TBI and OzENTER-TBI. Intensive Care Med. 2022;48(12):1709–25. 10.1007/s00134-022-06884-x. 36264365 10.1007/s00134-022-06884-x PMC9705485

[44] Hong WP, Hong KJ, Shin SD, Song KJ, Kim TH, Park JH, et al. Association of Flow Rate of Prehospital Oxygen Administration and clinical outcomes in severe traumatic brain Injury. J Clin Med. 2021;10(18). 10.3390/jcm10184097. 10.3390/jcm10184097 PMC8468196 34575206

[45] Sun W, Wang L, Yuan S, Liu R, Song P, Che W, et al. Association between Percutaneous Oxygen Saturation and Mortality of patients with mild traumatic brain Injury at ICU admission: an analysis of the MIMIC-III database. Adv Ther. 2023;40(6):2773–83. 10.1007/s12325-023-02499-w. 37084160 10.1007/s12325-023-02499-w

