# Supplementary material for: Dose-dependent association of hyperoxia and decreased favorable outcomes in mechanically ventilated patients with traumatic brain injury, a retrospective cohort study
Source: Eur J Trauma Emerg Surg. 2025 Jan 24;51(1):75. doi: 10.1007/s00068-024-02730-5 (PMC11761995; doi:10.1007/s00068-024-02730-5)

Table S1. Patient characteristics and univariable analysis for in-hospital mortality, all patients

patient characteristics	survived (n=198)	deceased (n=92)	p-value
age [years]	51.75 ± 20.02	64.22 ± 20.59	**< 0.001**
Charlson Comorbidity Index	1.0 [4.0]	4.0 [4.0]	**< 0.001**
Glasgow Coma Score	7.5 [9.75]	6.0 [10.0]	0.488
pupillary reactivity to light			**0.035**
both reactive	145 (73.2)	51 (55.4)	
one reactive	27 (13.6)	15 (16.3)	
none reactive	23 (11.6)	19 (20.7)	
unknown	3 (1.5)	7 (7.6)	
Abbreviated Injury Scale for head	4.0 [1.0]	5.0 [1.0]	**< 0.001**
Rotterdam-CT-Score	3.0 [1.0]	4.0 [2.0]	**< 0.001**
Simplified Acute Physiology Score II	44.0 [19.5]	53.5 [18.0]	**< 0.001**
**on day 1**			
mean PaO_2 [mmHg]	98.05 ± 21.12	100.92 ± 27.46	0.338
PaO_2 integral above 80 mmHg [mmHg/d]	20.88 ± 19.18	23.87 ± 25.69	0.278
PaO_2 integral above 100 mmHg [mmHg/d]	11.05 ± 15.05	13.80 ± 22.36	0.228
PaO_2 integral above 120 mmHg [mmHg/d]	6.56 ± 11.87	8.91 ± 19.66	0.302
PaO_2 integral above 150 mmHg [mmHg/d]	3.82 ± 9.11	5.60 ± 16.80	0.353
**admission to day 3**			
mean PaO_2 [mmHg]	90.96 ± 13.27	89.78 ± 15.17	0.508
PaO_2 integral above 80 mmHg [mmHg/d]	14.02 ± 11.10	13.84 ± 12.37	0.904
PaO_2 integral above 100 mmHg [mmHg/d]	5.62 ± 6.89	6.43 ± 9.16	0.410
PaO_2 integral above 120 mmHg [mmHg/d]	2.96 ± 4.85	4.20 ± 7.88	0.176
PaO_2 integral above 150 mmHg [mmHg/d]	1.66 ± 3.66	2.80 ± 6.63	0.134
**admission to day 7**			
mean PaO_2 [mmHg]	87.70 ± 10.37	94.55 ± 40.15	0.131
PaO_2 integral above 80 mmHg [mmHg/d]	10.95 ± 8.09	18.83 ± 38.46	0.070
PaO_2 integral above 100 mmHg [mmHg/d]	3.76 ± 4.17	12.06 ± 35.80	**0.039**
PaO_2 integral above 120 mmHg [mmHg/d]	1.88 ± 2.64	10.00 ± 33.31	**0.030**
PaO_2 integral above 150 mmHg [mmHg/d]	1.06 ± 1.92	8.27 ± 29.80	**0.032**
**admission to day 14**			
mean PaO_2 [mmHg]	86.20 ± 8.54	96.45 ± 46.72	0.105
PaO_2 integral above 80 mmHg [mmHg/d]	9.43 ± 6.44	20.70 ± 45.13	0.065
PaO_2 integral above 100 mmHg [mmHg/d]	2.82 ± 2.89	14.15 ± 42.00	**0.047**
PaO_2 integral above 120 mmHg [mmHg/d]	1.32 ± 1.76	11.78 ± 39.01	**0.048**
PaO_2 integral above 150 mmHg [mmHg/d]	0.76 ± 1.36	9.75 ± 34.61	0.055

Data are given as numbers and percentage in parentheses, mean ± standard deviation or median and interquartile range in brackets, as applicable. PaO_2 = arterial partial pressure of oxygen.

Table S2. Patient characteristics and univariable analysis for GOS after 3-6 months, all patients

**patient characteristics**	**favorable (n=72)**	**unfavorable (n=160)**	**p-value**
patients lost for follow-up: 58			
age [years]	45.08 ± 16.61	61.57 ± 20.84	**< 0.001**
Charlson Comorbidity Index	1.0 [1.0]	3.0 [5.0]	**< 0.001**
Glasgow Coma Score	8.5 [9.0]	7.0 [10.0]	0.206
pupillary reactivity to light			0.125
both reactive	56 (77.8)	99 (61.9)	
one reactive	7 (9.7)	27 (16.9)	
none reactive	8 (11.1)	25 (15.6)	
unknown	1 (1.4)	9 (5.6)	
Abbreviated Injury Scale for head	4.0 [1.0]	5.0 [1.0]	**0.021**
Rotterdam-CT-Score	3.0 [1.0]	4.0 [2.0]	**< 0.001**
Simplified Acute Physiology Score II	41.0 [22.0]	51.0 [18.25]	**< 0.001**
**on day 1**			
mean PaO_2 [mmHg]	97.41 ± 17.57	99.89 ± 24.81	0.392
PaO_2 integral above 80 mmHg [mmHg/d]	19.87 ± 15.91	22.73 ± 23.12	0.283
PaO_2 integral above 100 mmHg [mmHg/d]	9.51 ± 11.73	12.87 ± 19.71	0.123
PaO_2 integral above 120 mmHg [mmHg/d]	5.12 ± 8.91	8.08 ± 17.08	0.090
PaO_2 integral above 150 mmHg [mmHg/d]	2.78 ± 6.87	4.97 ± 14.32	0.122
**admission to day 3**			
mean PaO_2 [mmHg]	91.36 ± 12.43	90.44 ± 14.04	0.633
PaO_2 integral above 80 mmHg [mmHg/d]	14.01 ± 10.41	13.97 ± 11.59	0.983
PaO_2 integral above 100 mmHg [mmHg/d]	4.95 ± 6.14	6.27 ± 8.20	0.225
PaO_2 integral above 120 mmHg [mmHg/d]	2.39 ± 4.13	3.81 ± 6.76	0.053
PaO_2 integral above 150 mmHg [mmHg/d]	1.29 ± 3.17	2.38 ± 5.57	0.063
**admission to day 7**			
mean PaO_2 [mmHg]	89.01 ± 11.02	91.49 ± 30.39	0.378
PaO_2 integral above 80 mmHg [mmHg/d]	12.06 ± 8.92	15.15 ± 29.02	0.236
PaO_2 integral above 100 mmHg [mmHg/d]	4.01 ± 4.98	8.27 ± 26.82	0.062
PaO_2 integral above 120 mmHg [mmHg/d]	1.92 ± 3.12	6.31 ± 24.94	**0.036**
PaO_2 integral above 150 mmHg [mmHg/d]	1.10 ± 2.25	4.97 ± 22.29	**0.037**
**admission to day 14**			
mean PaO_2 [mmHg]	86.97 ± 9.22	91.29 ± 32.44	0.172
PaO_2 integral above 80 mmHg [mmHg/d]	10.07 ± 7.20	14.75 ± 31.31	0.116
PaO_2 integral above 100 mmHg [mmHg/d]	2.97 ± 3.45	8.03 ± 29.06	0.059
PaO_2 integral above 120 mmHg [mmHg/d]	1.29 ± 1.79	6.12 ± 26.97	0.050
PaO_2 integral above 150 mmHg [mmHg/d]	0.74 ± 1.29	4.86 ± 23.90	0.059

Data are given as numbers and percentage in parentheses, mean ± standard deviation or median and interquartile range in brackets, as applicable. PaO_2 = arterial partial pressure of oxygen.

Table S3. Patient characteristics and univariable analysis for GOS at discharge, all patients

patient characteristics	favorable (n=13)	unfavorable (n=277)	p-value
age [years]	45.23 ± 22.74	56.19 ± 20.82	0.066
Charlson Comorbidity Index	0.0 [2.5]	2.0 [4.0]	0.060
Glasgow Coma Score	13.5 [8.25]	7.0 [9.0]	**0.011**
pupillary reactivity to light			0.748
both reactive	10 (76.9)	186 (67.1)	
one reactive	2 (15.4)	40 (14.4)	
none reactive	1 (7.7)	41 (14.8)	
unknown	0 (0.0)	10 (3.6)	
Abbreviated Injury Scale for head	4.0 [1.0]	5.0 [1.0]	**0.012**
Rotterdam-CT-Score	3.0 [0.5]	3.0 [1.0]	0.281
Simplified Acute Physiology Score II	35.0 [19.0]	48.0 [19.0]	**0.003**
**on day 1**			
mean PaO_2 [mmHg]	92.89 ± 14.60	99.24 ± 23.60	0.160
PaO_2 integral above 80 mmHg [mmHg/d]	16.31 ± 14.44	22.09 ± 21.70	0.191
PaO_2 integral above 100 mmHg [mmHg/d]	7.63 ± 11.98	12.12 ± 17.90	0.372
PaO_2 integral above 120 mmHg [mmHg/d]	4.63 ± 10.66	7.43 ± 14.94	0.506
PaO_2 integral above 150 mmHg [mmHg/d]	3.15 ± 8.92	4.44 ± 12.20	0.707
**admission to day 3**			
mean PaO_2 [mmHg]	87.44 ± 10.95	90.74 ± 14.00	0.404
PaO_2 integral above 80 mmHg [mmHg/d]	12.05 ± 8.94	14.05 ± 11.58	0.540
PaO_2 integral above 100 mmHg [mmHg/d]	4.76 ± 6.55	5.93 ± 7.72	0.591
PaO_2 integral above 120 mmHg [mmHg/d]	2.81 ± 5.05	3.37 ± 6.03	0.745
PaO_2 integral above 150 mmHg [mmHg/d]	1.77 ± 3.67	2.03 ± 4.86	0.849
**admission to day 7**			
mean PaO_2 [mmHg]	84.40 ± 9.53	89.90 ± 24.10	0.406
PaO_2 integral above 80 mmHg [mmHg/d]	9.17 ± 6.32	13.49 ± 22.69	0.495
PaO_2 integral above 100 mmHg [mmHg/d]	2.86 ± 3.02	6.38 ± 20.53	0.537
PaO_2 integral above 120 mmHg [mmHg/d]	1.64 ± 2.18	4.41 ± 19.00	0.600
PaO_2 integral above 150 mmHg [mmHg/d]	1.00 ± 1.56	3.30 ± 16.95	0.625
**admission to day 14**			
mean PaO_2 [mmHg]	83.96 ± 11.18	88.84 ± 24.66	0.555
PaO_2 integral above 80 mmHg [mmHg/d]	8.42 ± 8.85	12.27 ± 23.54	0.624
PaO_2 integral above 100 mmHg [mmHg/d]	2.87 ± 3.97	5.64 ± 21.53	0.700
PaO_2 integral above 120 mmHg [mmHg/d]	1.02 ± 1.18	3.93 ± 19.92	0.662
PaO_2 integral above 150 mmHg [mmHg/d]	0.63 ± 0.89	3.00 ± 17.63	0.688

Data are given as numbers and percentage in parentheses, mean ± standard deviation or median and interquartile range in brackets, as applicable. PaO_2 = arterial partial pressure of oxygen.

Table S4. Patient characteristics and univariable analysis for in-hospital mortality, sensitivity analysis without palliative care patients (n=228)

patient characteristics	survived (n=198)	deceased (n=30)	p-value
age [years]	51.75 ± 20.02	51.53 ± 19.55	**< 0.001**
Charlson Comorbidity Index	1.0 [4.0]	1.0 [3.0]	**< 0.001**
Glasgow Coma Score	7.5 [9.75]	5.0 [7.75]	0.488
pupillary reactivity to light			**0.035**
both reactive	145 (73.2)	14 (46.7)	
one reactive	27 (13.6)	3 (10.0)	
none reactive	23 (11.6)	12 (40.0)	
unknown	3 (1.5)	1 (3.3)	
Abbreviated Injury Scale for head	4.0 [1.0]	5.0 [1.0]	**< 0.001**
Rotterdam-CT-Score	3.0 [1.0]	5.0 [1.5]	**< 0.001**
Simplified Acute Physiology Score II	44.0 [19.5]	55.0 [18.0]	**0.040**
**on day 1**			
mean PaO_2 [mmHg]	98.05 ± 21.12	106.29 ± 22.26	0.050
PaO_2 integral above 80 mmHg [mmHg/d]	20.87 ± 19.18	28.83 ± 20.52	**0.038**
PaO_2 integral above 100 mmHg [mmHg/d]	11.05 ± 15.05	17.79 ± 17.44	**0.027**
PaO_2 integral above 120 mmHg [mmHg/d]	6.56 ± 11.87	11.90 ± 14.72	0.067
PaO_2 integral above 150 mmHg [mmHg/d]	3.82 ± 9.11	7.24 ± 12.26	0.152
**admission to day 3**			
mean PaO_2 [mmHg]	90.96 ± 13.27	96.27 ± 13.74	**0.043**
PaO_2 integral above 80 mmHg [mmHg/d]	14.02 ± 11.07	19.02 ± 11.81	**0.023**
PaO_2 integral above 100 mmHg [mmHg/d]	5.62 ± 6.89	10.00 ± 9.33	**0.019**
PaO_2 integral above 120 mmHg [mmHg/d]	2.96 ± 4.85	7.06 ± 8.11	**0.011**
PaO_2 integral above 150 mmHg [mmHg/d]	1.66 ± 3.66	4.86 ± 6.88	**0.018**
**admission to day 7**			
mean PaO_2 [mmHg]	87.70 ± 10.37	115.04 ± 60.15	**0.019**
PaO_2 integral above 80 mmHg [mmHg/d]	10.95 ± 8.09	37.90 ± 58.73	**0.018**
PaO_2 integral above 100 mmHg [mmHg/d]	3.76 ± 4.17	28.58 ± 55.71	**0.021**
PaO_2 integral above 120 mmHg [mmHg/d]	1.88 ± 2.64	24.92 ± 52.11	**0.022**
PaO_2 integral above 150 mmHg [mmHg/d]	1.06 ± 1.92	21.10 ± 46.87	**0.026**
**admission to day 14**			
mean PaO_2 [mmHg]	86.20 ± 8.54	122.63 ± 74.07	**0.046**
PaO_2 integral above 80 mmHg [mmHg/d]	9.43 ± 6.44	46.14 ± 72.12	**0.040**
PaO_2 integral above 100 mmHg [mmHg/d]	2.82 ± 2.89	37.73 ± 67.74	**0.037**
PaO_2 integral above 120 mmHg [mmHg/d]	1.31 ± 1.76	33.46 ± 63.11	**0.039**
PaO_2 integral above 150 mmHg [mmHg/d]	0.76 ± 1.36	28.17 ± 56.42	**0.048**

Data are given as numbers and percentage in parentheses, mean ± standard deviation or median and interquartile range in brackets, as applicable. PaO_2 = arterial partial pressure of oxygen.

Table S5. Patient characteristics and univariable analysis for GOS after 3-6 months, sensitivity analysis without palliative care patients (n=228)

**patient characteristics**	**favorable (n=72)**	**unfavorable (n=98)**	**p-value**
patients lost for follow-up: 58			
age [years]	45.08 ± 16.61	55.85 ± 20.49	**< 0.001**
Charlson Comorbidity Index	1.0 [1.0]	2.0 [4.0]	**< 0.001**
Glasgow Coma Score	8.5 [9.0]	7.0 [8.0]	0.155
pupillary reactivity to light			0.160
both reactive	56 (77.8)	62 (63.3)	
one reactive	7 (9.7)	15 (15.3)	
none reactive	8 (11.1)	18 (18.4)	
unknown	1 (1.4)	3 (3.1)	
Abbreviated Injury Scale for head	4.0 [1.0]	5.0 [1.0]	0.087
Rotterdam-CT-Score	3.0 [1.0]	4.0 [2.0]	**< 0.001**
Simplified Acute Physiology Score II	41.0 [22.0]	50.0 [17.50]	**< 0.001**
**on day 1**			
mean PaO_2 [mmHg]	97.41 ± 17.57	100.92 (21.59)	0.247
PaO_2 integral above 80 mmHg [mmHg/d]	19.87 ± 15.91	23.57 (19.94)	0.184
PaO_2 integral above 100 mmHg [mmHg/d]	9.51 ± 11.73	13.40 (16.44)	0.076
PaO_2 integral above 120 mmHg [mmHg/d]	5.12 ± 8.91	8.50 (13.71)	0.055
PaO_2 integral above 150 mmHg [mmHg/d]	2.78 ± 6.87	5.10 (11.00)	0.094
**admission to day 3**			
mean PaO_2 [mmHg]	91.36 ± 12.43	92.81 ± 13.01	0.466
PaO_2 integral above 80 mmHg [mmHg/d]	14.01 ± 10.41	15.64 ± 11.12	0.336
PaO_2 integral above 100 mmHg [mmHg/d]	4.95 ± 6.14	7.27 ± 7.82	**0.032**
PaO_2 integral above 120 mmHg [mmHg/d]	2.39 ± 4.13	4.45 ± 6.29	**0.011**
PaO_2 integral above 150 mmHg [mmHg/d]	1.29 ± 3.17	2.75 ± 5.07	**0.022**
**admission to day 7**			
mean PaO_2 [mmHg]	89.01 ± 11.02	96.14 ± 36.00	0.068
PaO_2 integral above 80 mmHg [mmHg/d]	12.06 ± 8.92	19.03 ± 34.97	0.061
PaO_2 integral above 100 mmHg [mmHg/d]	4.01 ± 4.98	11.32 ± 33.69	**0.032**
PaO_2 integral above 120 mmHg [mmHg/d]	1.92 ± 3.12	8.92 ± 30.49	**0.026**
PaO_2 integral above 150 mmHg [mmHg/d]	1.10 ± 2.25	7.15 ± 27.30	**0.031**
**admission to day 14**			
mean PaO_2 [mmHg]	86.97 ± 9.22	94.83 ± 37.95	0.069
PaO_2 integral above 80 mmHg [mmHg/d]	10.07 ± 7.20	17.77 ± 37.02	0.064
PaO_2 integral above 100 mmHg [mmHg/d]	2.97 ± 3.45	10.57 ± 34.66	**0.048**
PaO_2 integral above 120 mmHg [mmHg/d]	1.29 ± 1.79	8.44 ± 32.20	**0.044**
PaO_2 integral above 150 mmHg [mmHg/d]	0.74 ± 1.29	6.80 ± 28.56	0.054

Data are given as numbers and percentage in parentheses, mean ± standard deviation or median and interquartile range in brackets, as applicable. PaO_2 = arterial partial pressure of oxygen.

Table S6. Patient characteristics and univariable analysis for in-hospital mortality, sensitivity analysis without patients with severe chest injury (n=275)

patient characteristics	survived (n=187)	deceased (n=88)	p-value
age [years]	52.50 ± 20.03	65.49 ± 19.86	< **0.001**
Charlson Comorbidity Index	1.0 [4.0]	4.0 [4.0]	< **0.001**
Glasgow Coma Score	8.0 [9.0]	7.0 [10.0]	0.537
pupillary reactivity to light			0.149
both reactive	135 (72.2)	50 (56.8)	
one reactive	26 (13.9)	15 (17.0)	
none reactive	23 (12.3)	16 (18.2)	
unknown	3 (1.6)	7 (8.0)	
Abbreviated Injury Scale for head	4.0 [1.0]	5.0 [1.0]	< **0.001**
Rotterdam-CT-Score	3.0 [1.0]	4.0 [2.0]	< **0.001**
Simplified Acute Physiology Score II	44.0 [19.0]	53.0 [17.0]	< **0.001**
**on day 1**			
mean PaO_2 [mmHg]	98.30 ± 21.42	101.00 ± 28.00	0.389
PaO_2 integral above 80 mmHg [mmHg/d]	21.13 ± 19.49	24.01 ± 26.20	0.319
PaO_2 integral above 100 mmHg [mmHg/d]	11.31 ± 15.35	14.00 ± 22.78	0.260
PaO_2 integral above 120 mmHg [mmHg/d]	6.76 ± 12.13	9.02 ± 20.03	0.341
PaO_2 integral above 150 mmHg [mmHg/d]	3.97 ± 9.34	5.66 ± 17.14	0.398
**admission to day 3**			
mean PaO_2 [mmHg]	90.91 ± 13.49	89.85 ± 15.50	0.571
PaO_2 integral above 80 mmHg [mmHg/d]	14.02 ± 11.23	13.96 ± 12.65	0.966
PaO_2 integral above 100 mmHg [mmHg/d]	5.68 ± 6.98	6.53 ± 9.35	0.406
PaO_2 integral above 120 mmHg [mmHg/d]	3.01 ± 4.92	4.26 ± 8.04	0.188
PaO_2 integral above 150 mmHg [mmHg/d]	1.71 ± 3.73	2.85 ± 6.77	0.149
**admission to day 7**			
mean PaO_2 [mmHg]	87.59 ± 10.56	93.10 ± 37.69	0.207
PaO_2 integral above 80 mmHg [mmHg/d]	10.91 ± 8.25	17.41 ± 36.00	0.119
PaO_2 integral above 100 mmHg [mmHg/d]	3.78 ± 4.25	10.71 ± 33.47	0.072
PaO_2 integral above 120 mmHg [mmHg/d]	1.91 ± 2.69	8.74 ± 31.21	0.057
PaO_2 integral above 150 mmHg [mmHg/d]	1.09 ± 1.97	7.17 ± 28.01	0.059
**admission to day 14**			
mean PaO_2 [mmHg]	86.08 ± 8.70	97.35 ± 47.85	0.091
PaO_2 integral above 80 mmHg [mmHg/d]	9.39 ± 6.55	21.54 ± 46.24	0.060
PaO_2 integral above 100 mmHg [mmHg/d]	2.83 ± 2.94	14.83 ± 43.07	**0.046**
PaO_2 integral above 120 mmHg [mmHg/d]	1.34 ± 1.80	12.36 ± 40.02	**0.048**
PaO_2 integral above 150 mmHg [mmHg/d]	0.78 ± 1.39	10.25 ± 35.51	0.055

Data are given as numbers and percentage in parentheses, mean ± standard deviation or median and interquartile range in brackets, as applicable. PaO_2 = arterial partial pressure of oxygen.

Table S7. Patient characteristics and univariable analysis for GOS after 3-6 months, sensitivity analysis without patients with severe chest injury (n=275)

patient characteristics	favorable (n=67)	unfavorable (n=152)	p-value
patients lost for follow-up: 56			
age [years]	45.96 ± 16.73	62.45 ± 20.50	**< 0.001**
Charlson Comorbidity Index	1.0 [1.0]	3.5 [4.0]	**< 0.001**
Glasgow Coma Score	9.0 [9.0]	7.0 [10.0]	0.220
pupillary reactivity to light			0.255
both reactive	51 (76.1)	95 (62.5)	
one reactive	7 (10.4)	26 (17.1)	
none reactive	8 (11.9)	22 (14.5)	
unknown	1 (1.5)	9 (5.9)	
Abbreviated Injury Scale for head	4.0 [1.0]	5.0 [1.0]	**0.012**
Rotterdam-CT-Score	3.0 [1.0]	4.0 [2.0]	**< 0.001**
Simplified Acute Physiology Score II	41.0 [22.0]	50.0 [19.0]	**< 0.001**
**on day 1**			
mean PaO_2 [mmHg]	97.89 ± 17.99	100.01 ± 25.30	0.489
PaO_2 integral above 80 mmHg [mmHg/d]	20.39 ± 16.28	22.86 ± 23.60	0.444
PaO_2 integral above 100 mmHg [mmHg/d]	10.02 ± 11.98	12.95 ± 20.14	0.190
PaO_2 integral above 120 mmHg [mmHg/d]	5.48 ± 9.15	8.21 ± 17.45	0.137
PaO_2 integral above 150 mmHg [mmHg/d]	2.99 ± 7.08	5.08 ± 14.66	0.162
**admission to day 3**			
mean PaO_2 [mmHg]	91.61 ± 12.72	90.34 ± 14.33	0.538
PaO_2 integral above 80 mmHg [mmHg/d]	14.30 ± 10.62	13.92 ± 11.81	0.824
PaO_2 integral above 100 mmHg [mmHg/d]	5.19 ± 6.28	6.26 ± 8.34	0.352
PaO_2 integral above 120 mmHg [mmHg/d]	2.55 ± 4.24	3.82 ± 6.88	0.100
PaO_2 integral above 150 mmHg [mmHg/d]	1.38 ± 3.27	2.41 ± 5.68	0.098
**admission to day 7**			
mean PaO_2 [mmHg]	89.13 ± 11.23	90.60 ± 28.64	0.689
PaO_2 integral above 80 mmHg [mmHg/d]	12.22 ± 9.11	14.31 ± 27.24	0.547
PaO_2 integral above 100 mmHg [mmHg/d]	4.16 ± 5.12	7.52 ± 25.09	0.128
PaO_2 integral above 120 mmHg [mmHg/d]	2.01 ± 3.21	5.64 ± 23.37	0.072
PaO_2 integral above 150 mmHg [mmHg/d]	1.16 ± 2.32	4.40 ± 20.95	0.072
**admission to day 14**			
mean PaO_2 [mmHg]	86.95 ± 9.51	91.57 ± 33.34	0.168
PaO_2 integral above 80 mmHg [mmHg/d]	10.14 ± 7.42	15.06 ± 32.20	0.120
PaO_2 integral above 100 mmHg [mmHg/d]	3.09 ± 3.55	8.33 ± 29.90	0.065
PaO_2 integral above 120 mmHg [mmHg/d]	1.36 ± 1.84	6.40 ± 27.75	0.054
PaO_2 integral above 150 mmHg [mmHg/d]	0.79 ± 1.33	5.12 ± 24.59	0.061

Data are given as numbers and percentage in parentheses, mean ± standard deviation or median and interquartile range in brackets, as applicable. PaO_2 = arterial partial pressure of oxygen.

Table S8. Multivariable analysis: in-hospital mortality, all patients

oxygenation parameter	odds ratio	lower CI	upper CI	p-value
**on day 1**				
mean PaO_2 [mmHg]	1.011	0.995	1.027	0.190
PaO_2 integral above 80 mmHg [mmHg/d]	1.011	0.994	1.029	0.207
PaO_2 integral above 100 mmHg [mmHg/d]	1.011	0.990	1.033	0.291
PaO_2 integral above 120 mmHg [mmHg/d]	1.010	0.985	1.036	0.427
PaO_2 integral above 150 mmHg [mmHg/d]	1.007	0.975	1.040	0.688
**admission to day 3**				
mean PaO_2 [mmHg]	1.006	0.980	1.033	0.643
PaO_2 integral above 80 mmHg [mmHg/d]	1.013	0.982	1.046	0.417
PaO_2 integral above 100 mmHg [mmHg/d]	1.025	0.979	1.072	0.288
PaO_2 integral above 120 mmHg [mmHg/d]	1.037	0.980	1.096	0.211
PaO_2 integral above 150 mmHg [mmHg/d]	1.043	0.973	1.118	0.238
**admission to day 7**				
mean PaO_2 [mmHg]	1.014	0.995	1.032	0.144
PaO_2 integral above 80 mmHg [mmHg/d]	1.022	0.992	1.052	0.148
PaO_2 integral above 100 mmHg [mmHg/d]	1.044	0.977	1.116	0.207
PaO_2 integral above 120 mmHg [mmHg/d]	1.074	0.963	1.198	0.198
PaO_2 integral above 150 mmHg [mmHg/d]	1.092	0.952	1.266	0.244
**admission to day 14**				
mean PaO_2 [mmHg]	1.018	0.999	1.038	0.068
PaO_2 integral above 80 mmHg [mmHg/d]	1.026	0.998	1.055	0.070
PaO_2 integral above 100 mmHg [mmHg/d]	1.045	0.982	1.113	0.164
PaO_2 integral above 120 mmHg [mmHg/d]	1.060	0.961	1.169	0.244
PaO_2 integral above 150 mmHg [mmHg/d]	1.068	0.932	1.224	0.342

Separate binary logistic regression model for each oxygenation parameter, adjusted for: age, Charlson Comorbidity Index, initial Glasgow Coma Score, pupillary reactivity to light, Rotterdam-CT-Score, Abbreviated Injury Scale for head and Simplified Acute Physiology Score II without points for age and Glasgow Coma Score. CI = 95% confidence intervals; PaO_2 = arterial partial pressure of oxygen.

Table S9. Multivariable analysis: favorable GOS after 3-6 months, all patients

oxygenation parameter	odds ratio	lower CI	upper CI	p-value
**on day 1**				
mean PaO_2 [mmHg]	0.959	0.930	0.990	**0.009**
PaO_2 integral above 80 mmHg [mmHg/d]	0.955	0.923	0.988	**0.008**
PaO_2 integral above 100 mmHg [mmHg/d]	0.939	0.897	0.982	**0.006**
PaO_2 integral above 120 mmHg [mmHg/d]	0.923	0.871	0.978	**0.007**
PaO_2 integral above 150 mmHg [mmHg/d]	0.922	0.858	0.992	**0.029**
**admission to day 3**				
mean PaO_2 [mmHg]	0.969	0.931	1.009	0.129
PaO_2 integral above 80 mmHg [mmHg/d]	0.958	0.911	1.007	0.091
PaO_2 integral above 100 mmHg [mmHg/d]	0.897	0.819	0.983	**0.020**
PaO_2 integral above 120 mmHg [mmHg/d]	0.842	0.738	0.961	**0.011**
PaO_2 integral above 150 mmHg [mmHg/d]	0.832	0.705	0.981	**0.029**
**admission to day 7**				
mean PaO_2 [mmHg]	0.989	0.961	1.019	0.471
PaO_2 integral above 80 mmHg [mmHg/d]	0.984	0.944	1.025	0.431
PaO_2 integral above 100 mmHg [mmHg/d]	0.952	0.863	1.051	0.333
PaO_2 integral above 120 mmHg [mmHg/d]	0.891	0.749	1.060	0.194
PaO_2 integral above 150 mmHg [mmHg/d]	0.885	0.699	1.121	0.312
**admission to day 14**				
mean PaO_2 [mmHg]	0.988	0.964	1.012	0.331
PaO_2 integral above 80 mmHg [mmHg/d]	0.984	0.952	1.016	0.321
PaO_2 integral above 100 mmHg [mmHg/d]	0.973	0.913	1.038	0.408
PaO_2 integral above 120 mmHg [mmHg/d]	0.949	0.777	1.159	0.606
PaO_2 integral above 150 mmHg [mmHg/d]	0.950	0.788	1.144	0.586

Separate binary logistic regression model for each oxygenation parameter, adjusted for: age, Charlson Comorbidity Index, initial Glasgow Coma Score, pupillary reactivity to light, Rotterdam-CT-Score, Abbreviated Injury Scale for head and Simplified Acute Physiology Score II without points for age and Glasgow Coma Score. CI = 95% confidence intervals; PaO_2 = arterial partial pressure of oxygen.

Table S10. Multivariable analysis: favorable GOS after 3-6 months, sensitivity analysis without palliative care patients

oxygenation parameter	odds ratio	lower CI	upper CI	p-value
**on day 1**				
mean PaO_2 [mmHg]	0.966	0.938	0.996	**0.025**
PaO_2 integral above 80 mmHg [mmHg/d]	0.963	0.932	0.995	**0.023**
PaO_2 integral above 100 mmHg [mmHg/d]	0.948	0.908	0.991	**0.018**
PaO_2 integral above 120 mmHg [mmHg/d]	0.934	0.883	0.989	**0.020**
PaO_2 integral above 150 mmHg [mmHg/d]	0.925	0.860	0.995	**0.037**
**admission to day 3**				
mean PaO_2 [mmHg]	0.973	0.934	1.014	0.189
PaO_2 integral above 80 mmHg [mmHg/d]	0.961	0.914	1.011	0.125
PaO_2 integral above 100 mmHg [mmHg/d]	0.903	0.824	0.989	**0.027**
PaO_2 integral above 120 mmHg [mmHg/d]	0.855	0.749	0.975	**0.019**
PaO_2 integral above 150 mmHg [mmHg/d]	0.834	0.706	0.985	**0.033**
**admission to day 7**				
mean PaO_2 [mmHg]	0.988	0.957	1.019	0.438
PaO_2 integral above 80 mmHg [mmHg/d]	0.983	0.943	1.025	0.422
PaO_2 integral above 100 mmHg [mmHg/d]	0.949	0.857	1.052	0.317
PaO_2 integral above 120 mmHg [mmHg/d]	0.886	0.741	1.059	0.184
PaO_2 integral above 150 mmHg [mmHg/d]	0.871	0.683	1.111	0.267
**admission to day 14**				
mean PaO_2 [mmHg]	0.987	0.963	1.012	0.303
PaO_2 integral above 80 mmHg [mmHg/d]	0.984	0.953	1.015	0.295
PaO_2 integral above 100 mmHg [mmHg/d]	0.973	0.917	1.032	0.360
PaO_2 integral above 120 mmHg [mmHg/d]	0.941	0.749	1.181	0.598
PaO_2 integral above 150 mmHg [mmHg/d]	0.936	0.718	1.220	0.625

Separate binary logistic regression model for each oxygenation parameter, adjusted for: age, Charlson Comorbidity Index, initial Glasgow Coma Score, pupillary reactivity to light, Rotterdam-CT-Score, Abbreviated Injury Scale for head Scale and Simplified Acute Physiology Score II without points for age and initial Glasgow Coma Score. CI = 95% confidence intervals; PaO_2 = arterial partial pressure of oxygen.

Table S11. Multivariable analysis: favorable GOS after 3-6 months, sensitivity analysis without patients with severe chest injury

**oxygenation parameter**	**odds ratio**	**lower CI**	**upper CI**	**p-value**
**on day 1**				
mean PaO_2 [mmHg]	0.963	0.934	0.993	**0.017**
PaO_2 integral above 80 mmHg [mmHg/d]	0.959	0.927	0.992	**0.016**
PaO_2 integral above 100 mmHg [mmHg/d]	0.944	0.902	0.988	**0.013**
PaO_2 integral above 120 mmHg [mmHg/d]	0.928	0.875	0.984	**0.013**
PaO_2 integral above 150 mmHg [mmHg/d]	0.929	0.863	1.000	0.050
**admission to day 3**				
mean PaO_2 [mmHg]	0.972	0.93	1.012	0.163
PaO_2 integral above 80 mmHg [mmHg/d]	0.961	0.914	1.010	0.118
PaO_2 integral above 100 mmHg [mmHg/d]	0.905	0.826	0.991	**0.032**
PaO_2 integral above 120 mmHg [mmHg/d]	0.851	0.745	0.973	**0.018**
PaO_2 integral above 150 mmHg [mmHg/d]	0.845	0.716	0.996	**0.045**
**admission to day 7**				
mean PaO_2 [mmHg]	0.991	0.963	1.019	0.521
PaO_2 integral above 80 mmHg [mmHg/d]	0.985	0.947	1.026	0.471
PaO_2 integral above 100 mmHg [mmHg/d]	0.956	0.868	1.054	0.369
PaO_2 integral above 120 mmHg [mmHg/d]	0.898	0.754	1.070	0.230
PaO_2 integral above 150 mmHg [mmHg/d]	0.895	0.707	1.133	0.356
**admission to day 14**				
mean PaO_2 [mmHg]	0.988	0.964	1.012	0.314
PaO_2 integral above 80 mmHg [mmHg/d]	0.983	0.953	1.015	0.306
PaO_2 integral above 100 mmHg [mmHg/d]	0.974	0.918	1.033	0.385
PaO_2 integral above 120 mmHg [mmHg/d]	0.954	0.823	1.106	0.534
PaO_2 integral above 150 mmHg [mmHg/d]	0.944	0.796	1.121	0.513

Separate binary logistic regression model for each oxygenation parameter, adjusted for: age, Charlson Comorbidity Index, initial Glasgow Coma Score, pupillary reactivity to light, Rotterdam-CT-Score, Abbreviated Injury Scale for head and Simplified Acute Physiology Score II without points for age and initial Glasgow Coma Score. CI = 95% confidence intervals; PaO_2 = arterial partial pressure of oxygen.

Table S12. Discontinuation of mechanical ventilation during the first 14 days after admission

parameter [mmHg] n=237	Q1 (64-79) n=60	Q2 (79-85) n=59	Q3 (85-92) n=59	Q4 (92-322) n=59	p-value
extubation	14 (23)	17 (29)	20 (34)	10 (17)	0.178
death	18 (30)	17 (29)	4 (7)	17 (29)	**0.006**
extubation or death	32 (53)	34 (58)	24 (41)	27 (46)	0.253

Q1 to Q4 refer the quartiles obtained by categorization according to time-weighted mean arterial oxygen partial pressure after 14 days, numbers in parentheses depict the particular range in PaO_2 in mmHg. Separate Chi-square tests were done for each parameter. Data are given as numbers and percent in parentheses. Table only includes deaths up to day 14.

Table S13. Time-weighted mean oxygen partial pressures before and after extubation

Parameter	before extubation	after extubation	p-value
mean PaO_2 [mmHg]	88.8 ± 9.24	83.3 ± 11.92	**< 0.001**

Patients extubated alive before day 14 (n=62). Paired t-test reveals a statistically significant difference with both values being within a normoxic range. PaO_2 = arterial partial pressure of oxygen

Table S14. Cause of in-hospital mortality, grouped by arterial oxygen partial pressure during the first 24 hours

parameter [mmHg] n=279	Q1 (53-83) n=70	Q2 (83-95) n=70	Q3 (95-108) n=70	Q4 (108-268) n=69
organ failure despite intensive care therapy	0	0	1	2
brain death	4	5	10	8
withdrawal of care due to poor prognosis and presumed will of patient	16	17	14	10
total	20	23	25	20

Q1 to Q4 refer to the quartiles obtained by categorization according to time-weighted mean arterial oxygen partial pressure during the first day; Chi-square reveals no significant difference, p=0.405; data are given as numbers. Table includes all deaths during initial hospital therapy.

Table S15. Cause of in-hospital mortality, grouped by arterial oxygen partial pressure during the first 72 hours

parameter [mmHg] n=283	Q1 (61-81) n=71	Q2 (81-89) n=71	Q3 (89-99) n=71	Q4 (99-149) n=70
organ failure despite intensive care therapy	0	0	3	0
brain death	3	4	11	9
withdrawal of care due to poor prognosis and presumed will of patient	20	19	8	11
total	23	23	22	20

Q1 to Q4 refer to the quartiles obtained by categorization according to time-weighted mean arterial oxygen partial pressure during the first three days; Chi-square reveals statistically significant difference, p=0.006; data are given as numbers. Table includes all deaths during initial hospital therapy.

Fig. S1 Proportion of mechanically ventilated patients over time

Discontinuity within the first 14 days of ventilation due to extubation or death of the patient.

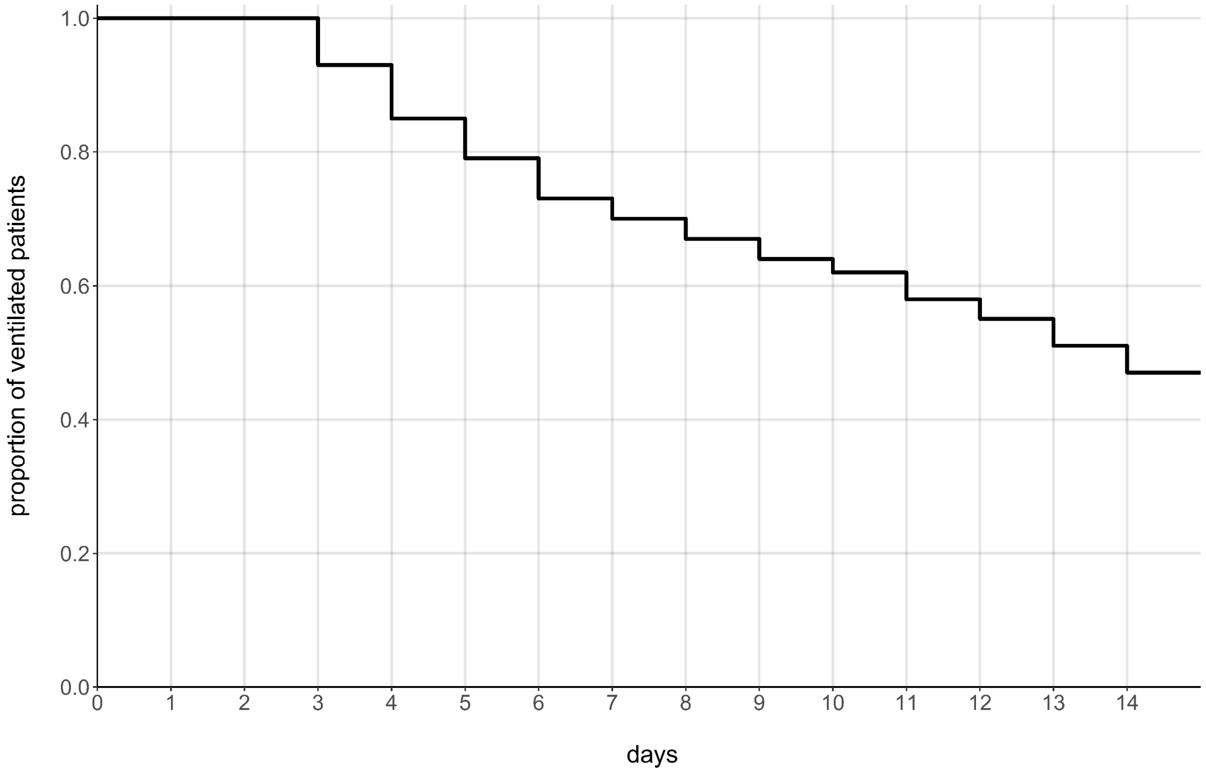

Fig. S2 Logistic regression model for in-hospital mortality, unadjusted

Logistic regression model for in-hospital mortality modelling time-weighted mean PaO_2 in mmHg as a restricted cubic spline with 3 knots. Black solid lines depict Odds Ratios in reference to the PaO_2 labelled with a vertical dashed line. Gray areas represent 95% confidence intervals.

A = on day one, reference: PaO_2 = 100 mmHg

B = admission to day 3, reference: PaO_2 = 95 mmHg

C = admission to day 7, reference: PaO_2 = 90 mmHg

D = admission to day 14, reference: PaO_2 = 88 mmHg

PaO_2 = arterial partial pressure of oxygen.

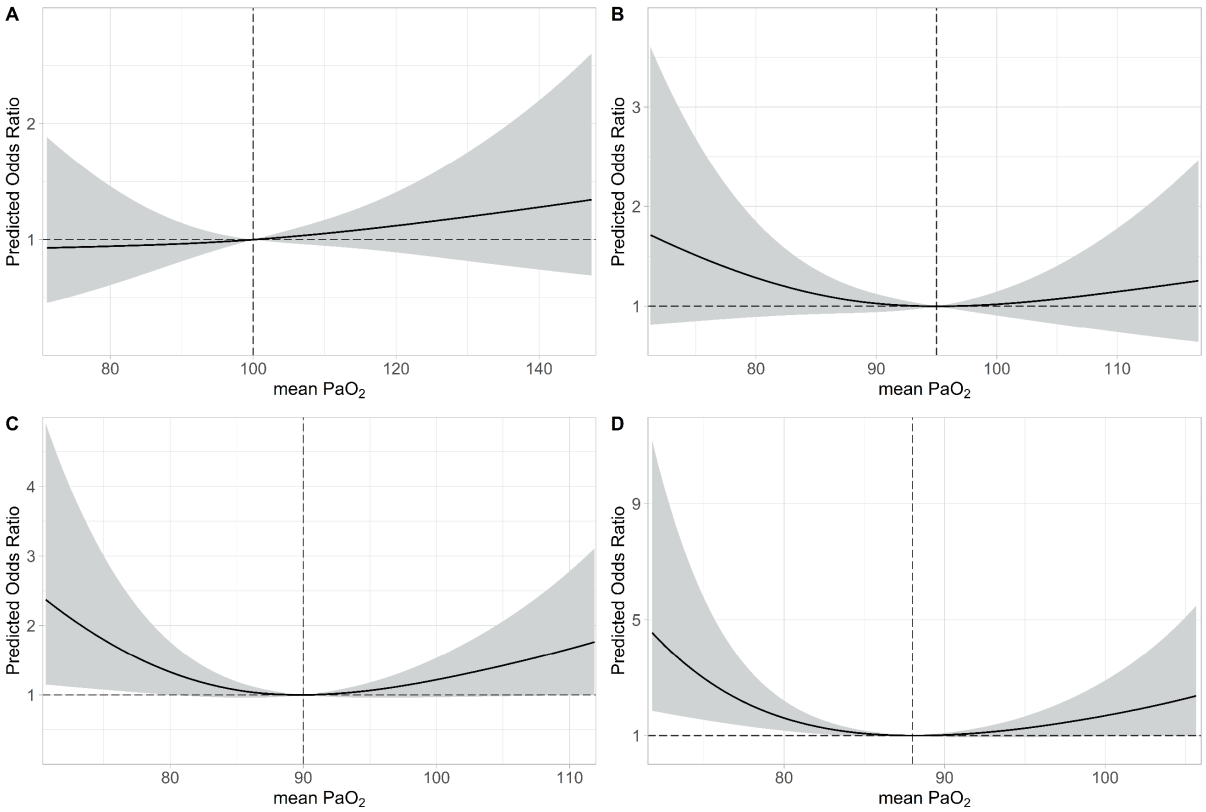

Fig. S3 Logistic regression model for unfavorable GOS after 3-6 months, unadjusted

Logistic regression model for an unfavorable GOS after 3-6 months, modelling time-weighted mean PaO_2 in mmHg as a restricted cubic spline with 3 knots. Black solid lines depict Odds Ratios in reference to the PaO_2 labelled with a vertical dashed line. Gray areas represent 95% confidence intervals.

A = on day one, reference: PaO_2 = 98 mmHg

B = admission to day 3, reference: PaO_2 = 95 mmHg

C = admission to day 7, reference: PaO_2 = 92 mmHg

D = admission to day 14, reference: PaO_2 = 89 mmHg

PaO_2 = arterial partial pressure of oxygen.

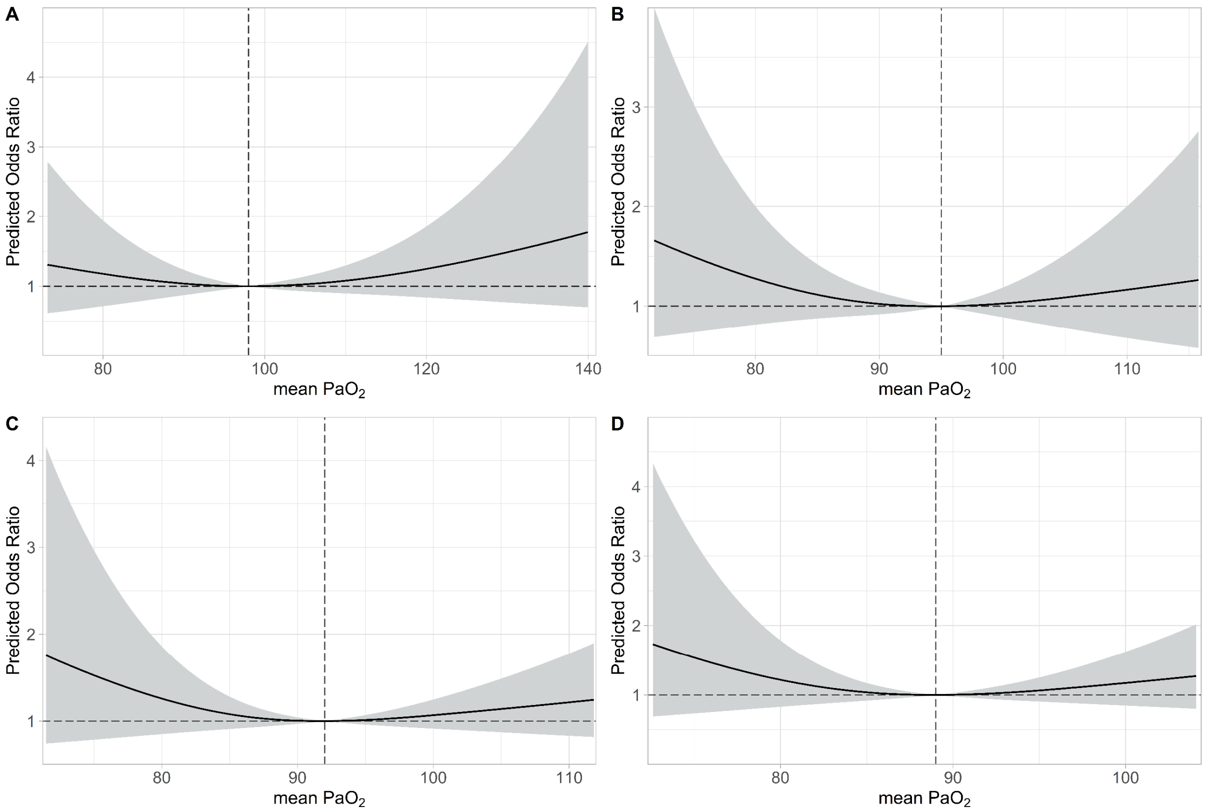

Fig. S4 Logistic regression model for in-hospital mortality, multivariable adjusted

Logistic regression model for in-hospital mortality modelling time-weighted mean PaO_2 in mmHg as a restricted cubic spline with 3 knots. Black solid lines depict Odds Ratios in reference to the PaO_2 labelled with a vertical dashed line. Gray areas represent 95% confidence intervals. Models were adjusted for: age, Charlson Comorbidity Index, initial Glasgow Coma Score, pupillary reactivity to light, Rotterdam-CT-Score, Abbreviated Injury Scale for head and Simplified Acute Physiology Score II without points for age and initial Glasgow Coma Score.

A = on day one, reference: PaO_2 = 114 mmHg

B = admission to day 3, reference: PaO_2 = 88 mmHg

C = admission to day 7, reference: PaO_2 = 88 mmHg

D = admission to day 14, reference: PaO_2 = 88 mmHg

PaO_2 = arterial partial pressure of oxygen.

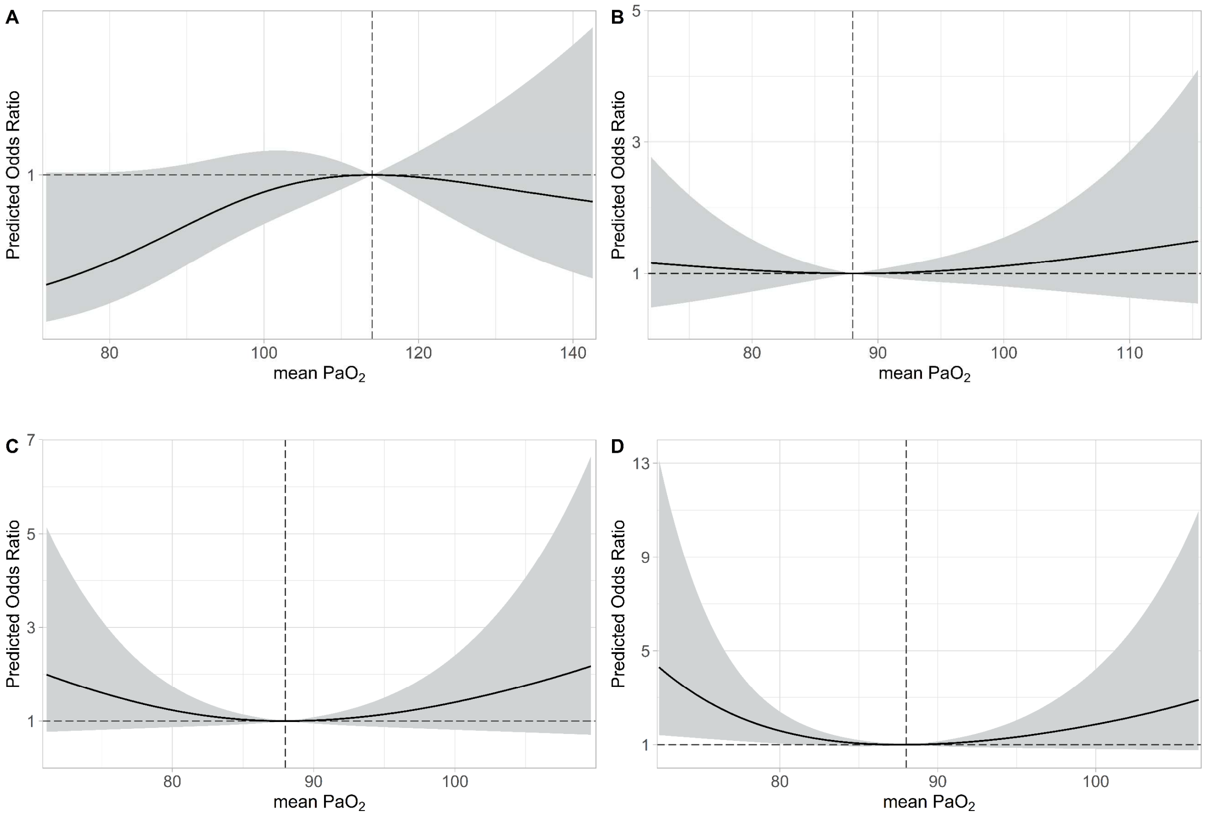

Fig. S5 Logistic regression model for unfavorable GOS after 3-6 months, multivariable adjusted

Logistic regression model for an unfavorable GOS after 3-6 months, modelling time-weighted mean PaO_2 in mmHg as a restricted cubic spline with 3 knots. Black solid lines depict Odds Ratios in reference to the PaO_2 labelled with a vertical dashed line. Gray areas represent 95% confidence intervals. Models were adjusted for: age, Charlson Comorbidity Index, initial Glasgow Coma Score, pupillary reactivity to light, Rotterdam-CT-Score, Abbreviated Injury Scale for head and Simplified Acute Physiology Score II without points for age and initial Glasgow Coma Score.

A = on day one, reference: PaO_2 = 100 mmHg

B = admission to day 3, reference: PaO_2 = 85 mmHg

C = admission to day 7, reference: PaO_2 = 88 mmHg

D = admission to day 14, reference: PaO_2 = 88 mmHg

PaO_2 = arterial partial pressure of oxygen.

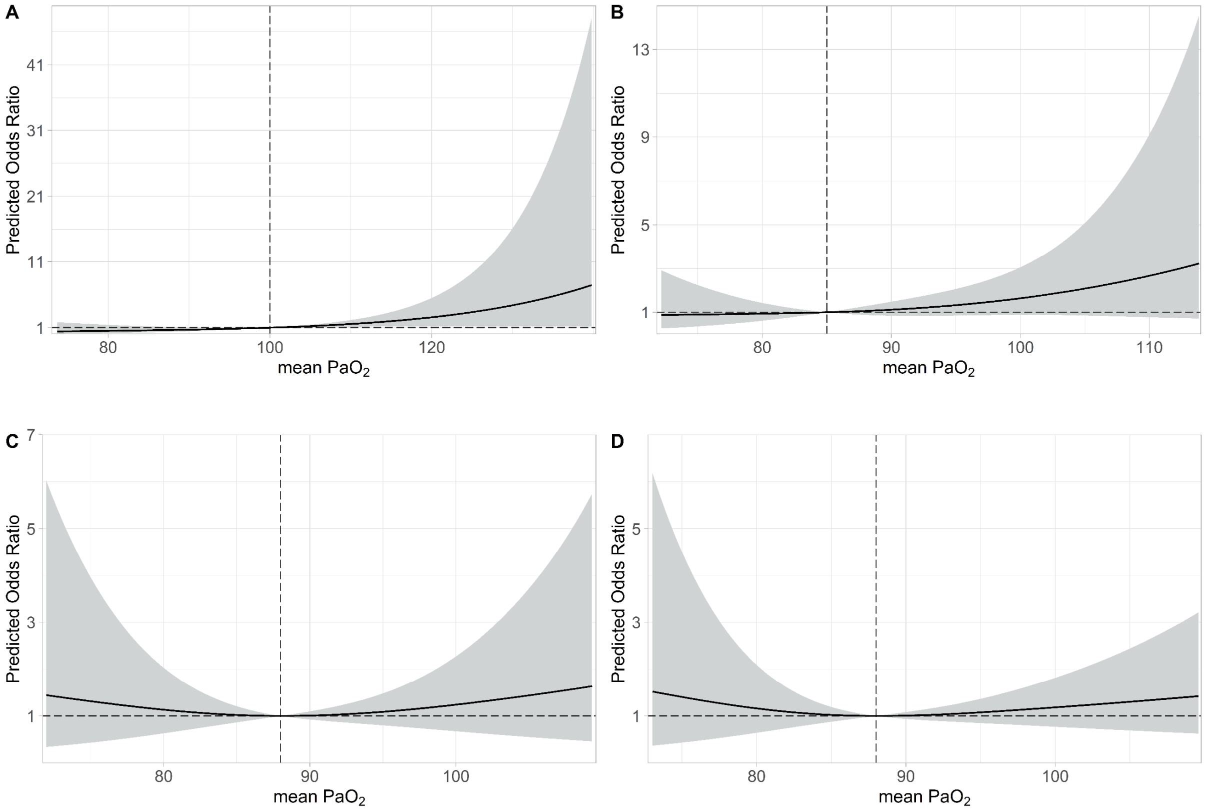

Fig. S6 Distribution of time-weighted mean PaO2 for days 1, 3, 7 and 14

Change of distribution of time-weighted mean PaO_2 over time.

Light gray = distribution of time-weighted mean PaO_2 at day 1.

Middle gray = distribution of time-weighted mean PaO_2 up to day 3.

Dark gray = distribution of time-weighted mean PaO_2 up to day 7.

Black = distribution of time-weighted mean PaO_2 until day 14.

PaO_2 = arterial partial pressure of oxygen.

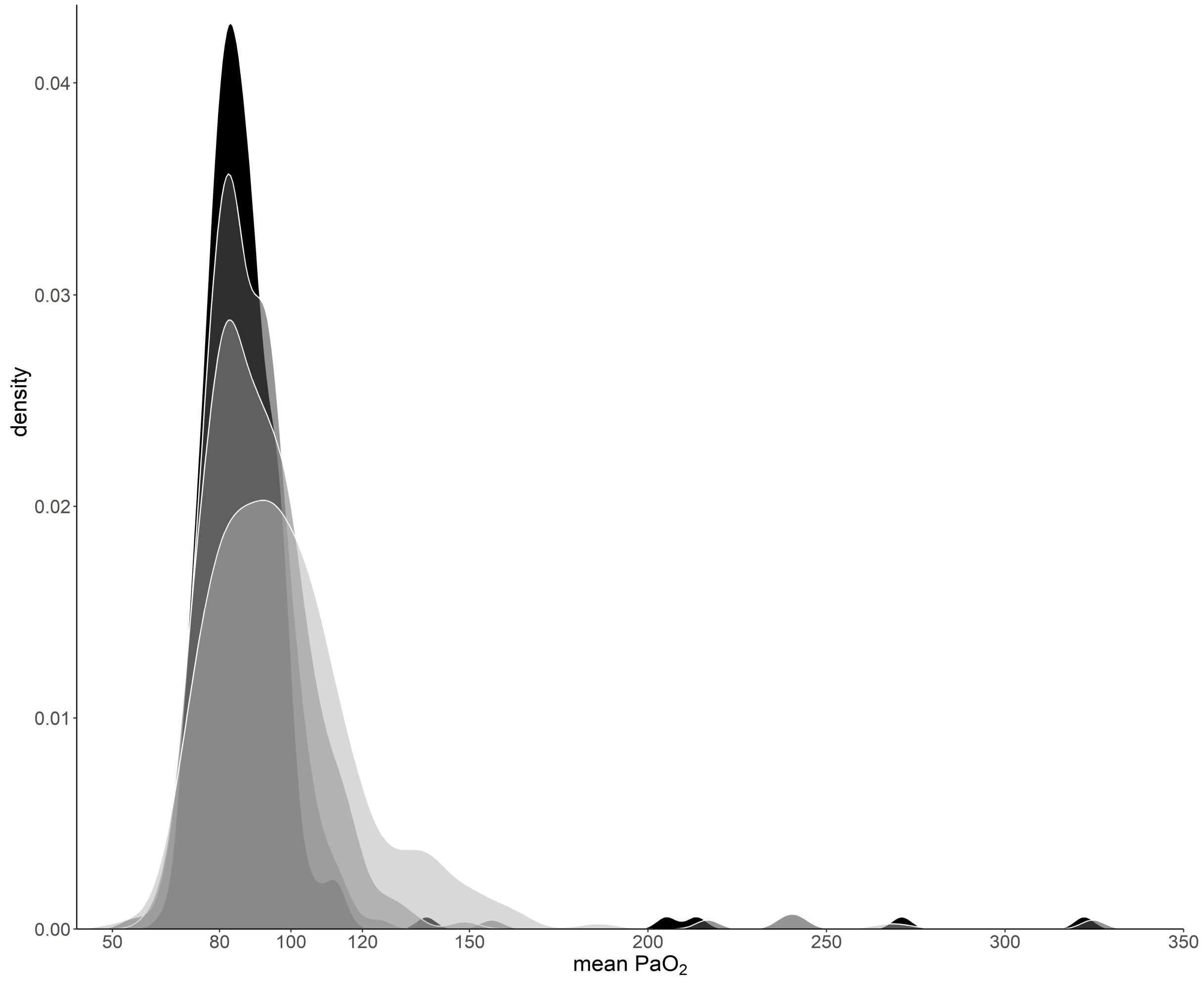

Supplement: Supplementary file 1 — Supplementary Material 1 [file 68_2024_2730_MOESM1_ESM.pdf]
